# Shedding Some Light over the Floral Metabolism by Arum Lily (*Zantedeschia aethiopica*) Spathe *De Novo* Transcriptome Assembly

**DOI:** 10.1371/journal.pone.0090487

**Published:** 2014-03-10

**Authors:** Elizabete de Souza Cândido, Gabriel da Rocha Fernandes, Sérgio Amorim de Alencar, Marlon Henrique e Silva Cardoso, Stella Maris de Freitas Lima, Vívian de Jesus Miranda, William Farias Porto, Diego Oliveira Nolasco, Nelson Gomes de Oliveira-Júnior, Aulus Estevão Anjos de Deus Barbosa, Robert Edward Pogue, Taia Maria Berto Rezende, Simoni Campos Dias, Octávio Luiz Franco

**Affiliations:** 1 Programa de Pós-Graduação em Ciências Genômicas e Biotecnologia, Universidade Católica de Brasília, Brasília-DF, Brazil; 2 Centro de Análises Proteômicas e Bioquímicas, Pós-Graduação em Ciências Genômicas e Biotecnologia, Universidade Católica de Brasília, Brasília-DF, Brazil; 3 Programa de Pós-Graduação em Biologia Animal, Universidade de Brasília, Brasília-DF, Brazil; 4 Curso de Física, Universidade Católica de Brasília, Brasília – DF, Brazil; 5 Curso de Odontologia, Universidade Católica de Brasília, Brasília – DF, Brazil; Shenzhen Institutes of Advanced Technology, China

## Abstract

*Zantedeschia aethiopica* is an evergreen perennial plant cultivated worldwide and commonly used for ornamental and medicinal purposes including the treatment of bacterial infections. However, the current understanding of molecular and physiological mechanisms in this plant is limited, in comparison to other non-model plants. In order to improve understanding of the biology of this botanical species, RNA-Seq technology was used for transcriptome assembly and characterization. Following *Z. aethiopica* spathe tissue RNA extraction, high-throughput RNA sequencing was performed with the aim of obtaining both abundant and rare transcript data. Functional profiling based on KEGG Orthology (KO) analysis highlighted contigs that were involved predominantly in genetic information (37%) and metabolism (34%) processes. Predicted proteins involved in the plant circadian system, hormone signal transduction, secondary metabolism and basal immunity are described here. *In silico* screening of the transcriptome data set for antimicrobial peptide (AMP) –encoding sequences was also carried out and three lipid transfer proteins (LTP) were identified as potential AMPs involved in plant defense. Spathe predicted protein maps were drawn, and suggested that major plant efforts are expended in guaranteeing the maintenance of cell homeostasis, characterized by high investment in carbohydrate, amino acid and energy metabolism as well as in genetic information.

## Introduction

Plants have evolved to create an extensive and sophisticated defense system against predators and pathogens. It is already known that plants respond to biotic and abiotic stress in a complex fashion, with these events being regulated by multiple signaling pathways showing a significant overlap between the gene expression patterns that can be induced in reaction to different stresses [1], [2]. Practically all plant organs have been studied with the aim of elucidating the defense system complexity, but just a few studies have been dedicated to the floral organs. In spite of this, floral tissues can be highly useful as resources for the development of new antimicrobial compounds for the benefit of human health and agribusiness [3]. Some reports have successfully portrayed the use of floral organs as potential antimicrobial sources, such as the defensins from tomato [4] and tobacco [5], and plant lipid transfer proteins (LTPs) from rice [6] that have been described with the capacity to improve plant antimicrobial resistance. Furthermore, the hormonal changes in response to abiotic and biotic stress have been broadly studied in plants such as Arabidopsis [2] and *Reaumuria soongorica* [7], highlighting the importance of interconnections between innate immunity and plant development.

The arum lily (*Zantedeschia aethiopica*) is an evergreen perennial plant from the Araceae family, and is known worldwide for ornamental purposes. However, in some African regions this plant has been commonly used in traditional medicine. The rhizomes are utilized as cataplasms for abscess and boil reduction and fresh leaves have been commonly used for asthma and bronchitis treatment and also for treatment of skin wounds [8]. By late 2012, 43 EST-derived simple sequence repeats (SSR) from *Z. aethiopica* had been elucidated, representing the first data set of polymorphic microsatellite markers for this genus [9]. Currently it is possible to access 4.394 EST sequences for this plant available through the National Center for Biotechnology Information (NCBI). In view of the lack of a complete genome sequence and the impossibility of acquiring these data for many eukaryotes, transcriptome characterization arises as an attractive alternative for gene discovery, helping to identify transcripts involved in several biological processes [10], [11]. *Z. aethiopica* has not yet had its genome elucidated, and knowledge about its molecular and physiological defense mechanisms is still limited, necessitating the pursuit of strategies such as transcriptome sequencing to enhance the study of this non-model plant. In this regard, next-generation high-throughput RNA sequencing (RNA-Seq) provides excellent tools for the discovery, profiling and quantification of RNA transcripts [12]. Due to the high degree of sequence coverage, this technology enables the identification not only of abundant transcripts, but also of rare ones, which is particularly useful for the study of the transcriptome of organisms that do not have reference genomes available [13], [14].

In this context, in order to characterize the molecular and physiological defense aspects of *Z. aethiopica*, without stress stimuli, a transcriptional profile analysis was performed. *In-silico* screening for predicted AMPs in the transcriptome data set was also carried out, with the aim of characterizing a wide variety of defense mechanisms using the biological information obtained. Our study identified several potential candidate transcripts which were predicted to be involved in plant-pathogen interactions, plant hormone signal transduction, and metabolic pathways.

## Results and Discussion

### Prospection of floral tissues with antimicrobial properties

Initially, a selection process involving ten different plant species was performed (Table 1), aiming to track down floral tissues with antimicrobial properties. Antibacterial assays against *E. coli* and *S. aureus* were carried out with protein-rich fractions from each of the ten species, by using a standard protein concentration of 200 µg mL^−1^ of each sample. Forty µg mL^−1^ of chloramphenicol was utilized as positive control (representing 100% bacterial growth inhibition) and sterile distilled water was used as negative control (representing 0% bacterial growth inhibition). As can be observed in Table 1, from all samples tested, *Z. aethiopica* spathe showed the highest antimicrobial activity against *E. coli* development, causing about 96% growth inhibition, though no activity against *S. aureus* was detected. Interestingly, the spadix showed no antimicrobial activity against bacteria, and this was also the case for rose (*Rosa* sp.) and carnation (*Dianthus caryophyllus*). For Madagascar periwinkle (*Catharanthus roseus*) 50% and 17% growth inhibition for *E. coli* and *S. aureus* was detected respectively. Paper flower (*Bouganvillea glabra*) caused only a small inhibition against *E. coli* with a value of 4%. Finally, the antimicrobial activities of orchid tree (*Bauhinia variegata*), oleander (*Nerium oleander*), daisy (*Bellis* sp.), lisianthus (*Lisianthus* sp.) and dwarf silk oak (*Grevillea banskii*) were not confirmed since these samples caused the formation of a granular precipitate, which constrains accurate absorbance measurements. No antibacterial activity has previously been described for *Z. aethiopica* tissues, though Lin et al. [15] showed an antifungal activity of a *Z. aethiopica* agglutinin which acts against the leaf mold *Fulvia fulva* in a manner similar to lectin, when expressed in *E. coli.* This gave an indication of the antimicrobial potential of this plant. Furthermore, antimicrobial activities of secondary metabolites have been described for *Rosa* sp. [16], [17], *D. caryophyllus* [18], [19], *C. roseus* [20], [21], *B. variegata* [22], *N. oleander* [23], *Bellis* [24] and *Grevillea* [25], however no floral tissues were used for these purposes.

**Table 1:** Evaluation of floral extracts antimicrobial potential against *E. coli* (ATCC8739) and *S. aureus* (ATCC29213).

Common Name	Species	Botanical Family	Structure	Bacterial Growth Inhibition (%)	
				*Escherichia coli* (ATCC8739)	*Staphylococcus aureus* (ATCC29213)
Madagascar periwinkle	*Catharanthus roseus*	Apocynaceae	Petals	50,27	17,21
Orchid tree	*Bauhinia variegata*	Fabaceae	Petals	NC	NC
Paper flower	*Bouganvillea glabra*	Nyctaginaceae	Bracts	4,30	0
Rose	*Rosa* sp.	Rosaceae	Petals	0	0
Oleander	*Nerium oleander*	Apocynaceae	Petals	NC	NC
Daisy	*Bellis* sp.	Asteraceae	Ligules	NC	NC
Lisianthus	*Lisianthus* sp.	Gentianaceae	Petals	NC	NC
Carnation	*Dianthus caryophyllus*	Caryophyllaceae	Petals	0	0
Dwarf silky oak	*Grevillea banskii*	Proteaceae	Inflorescence	NC	NC
Arum Lily	*Zantedeschia aethiopica*	Araceae	Spadix	0	0
Arum Lily	*Zantedeschia aethiopica*	Araceae	Spathe	96,28	0

Were used 200 μg mL^−1^ of total protein for all tissues tested. Chloramphenicol 40 µg mL^−1^ was used as a positive control growth inhibition and sterile distilled water was used as negative control. Bacterial growth was measured using spectrophotometry (595 nm).

NC: Antimicrobial activity not confirmed.

### RNA-Seq and transcriptome assembly for *Z. aethiopica*

Considering the results of the antimicrobial assays, *Z. aethiopica* presented the best potential as an antimicrobial source, since it showed the highest inhibition of *E. coli* growth. An agarose gel was performed to evaluate the quality of total RNA (Figure S1). In order to understand the basal defense system at the transcriptional level of *Z. aethiopica*, one sequencing lane was used on the Illumina HiSeq 2000 platform yielding a total of 91,218,320 paired-end reads. Furthermore, aiming to improve the accuracy and computational efficiency of the transcriptome assembly [26], data pre-processing was carried out by removing duplicate paired-end reads, and eliminating sequencing adaptors and low-quality reads using the Trimmomatic trimming tool [27]. The ‘seedMismatches’ parameter for adapter removal was set as 2, allowing a maximum mismatch of 2 nucleotides between adapter sequences and sequenced reads. Leading and trailing bases with quality below 3 were removed, as well as ‘N’ bases. A 4-base wide sliding window was set in order to scan the reads and cut when the average quality per base dropped below 15. All pre-processed reads below 36 bases long were removed. As a result of pre-processing, a total of 19,622,994 paired-end and 3,846,882 single-end high quality reads were obtained (Table 2). The high quality paired-end and single-end reads were then used for *de novo* transcriptome assembly using the Trinity software [28], resulting in a total of 83,578 contigs (N50: 1,600 bp; Minimum length: 201 bp; Maximum length: 16,583; Mean length: 818 bp). The high-throughput sequencing data generated by the Illumina Hi-Seq platform permits *de novo* transcriptome assemblies, improving our understanding of gene control and gene networks [29].

**Table 2:** Sequencing and assembly of *Zantedeschia aethiopica* transcriptome using Illumina HiSeq 2000.

	Number
Total paired-ends reads	91,218,320
Clean reads	24,469,876
Total of contigs	83,578
Total of predicted genes assigned	29,506

### Functional Profile by KEGG Orthology (KO) and pathway mapping

**Figure 1:**
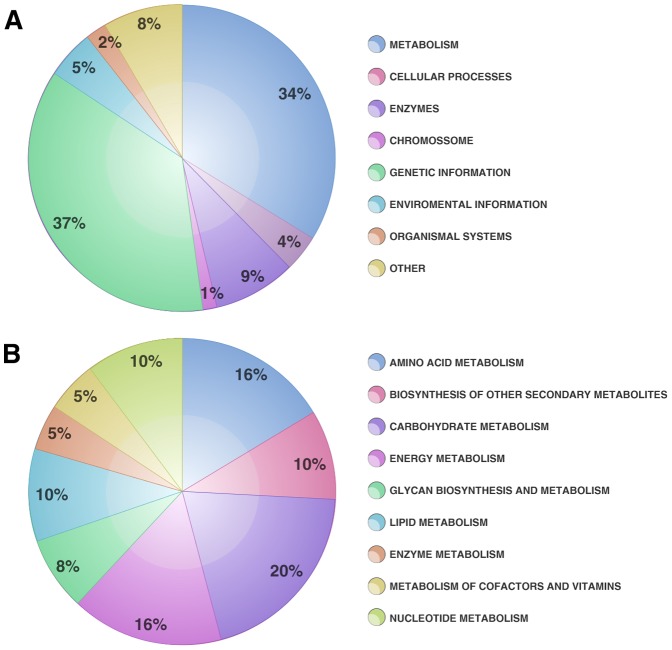
Categorization of *Z. aethiopica* spathe transcriptome into KEGG biological categories. A. Total KEGG biological categories contigs distribution; B. Metabolism biological category distribution of contigs percentage.

Initially, using GeneMark, 29,506 predicted genes were assigned and arbitrary nomenclature was designed to give names to the assembled contigs identified through the KEGG current database. Predicted genes were remapped using the TransDecoder prediction tool [30] for the purpose of cross-validate the GeneMark prediction. These results could be observed at a Venn diagram (Figure S2) as well as the gene coverage (Figure S3 and Table S3). In this data it is possible to remark that 2579 of the analyzed KOs were shared by both tools. Nevertheless only 294 KOs were covered just for GeneMark analysis and 628 KOs were covered just for TransDecoder analysis (Figure S2). In order to construct a reliable functional profile, only the genes covered by both methodologies were utilized, with few exceptions such as the KOs K122121 (Phy B), K12133 (LHY) and K13426 (WRKY 29), covered just for GeneMark prediction and that were explored at circadian systems and innate immunity approach detailed bellow. For functional profile generation, the transcriptome dataset provided by the *de novo* assembly was compared to the KEGG Orthology database. 2,870 KO assignments were obtained and these were divided into eight biological classes that can be visualized in Figure 1A. It is reasonable that the largest KO group was related to genetic information, given the enormous investment that plants devote to gene transcription control and capacity. These data corroborate data obtained from the *Arabidopsis* genome in which 1,500 transcription factors were observed [1]. Similar results were described for chickpea (*Cicer arietinum*) [31], bamboo (*Dendrocalamus latiflorus*) [29], safflower (*Carthamus tinctorius*) [32], orchid (*Erycina pusilla*) [33], and rapeseed (*Brassica napus*) [34] transcriptomes, in which the most highly represented classes were genetic information and metabolism. All of the above-mentioned studies demonstrated that contigs related to biosynthesis and metabolism of secondary metabolites, are relevant components of plant transcriptomes, occupying a prominent position in functional analysis datasets. Due to the clear importance of metabolism for cell maintenance and defense capacity, these biological classes were minutely analyzed, and are represented in Figure 1B. It was observed that the major portion of the contigs found in the metabolism class were dedicated to carbohydrate metabolism (20%), followed by energy (16%), amino acid metabolism (16%) and biosynthesis of secondary metabolites (10%). Data presented here are similar to those obtained for orchid [33] and rapeseed [34]. In both cases, transcripts analysis presented carbohydrate and amino acid metabolism and also secondary metabolite biosynthesis as the main representative categories. Also, it was observed nucleotide metabolism (10%) and lipid metabolism (10%). Nucleotide metabolism is one of the most important processes for all organisms' survival. In plants this is a similar situation and those processes represents a crucial role for plant metabolism and development. In these organisms the nucleotides can be produced from simple molecules including amino acids, CO_2_ or tetrahydrofolate and from 5-phosphoribosyl-1-pyrophosphate, or even derivate from nucleosides or nucleobases through salvage reactions [35], [36]. These molecules can be degraded into simple metabolites, which provides the recycling of phosphate, carbon, and nitrogen into the central metabolisms [35], [37], [38], making the nucleotide metabolism one of the most important process in plant survival. Furthermore, lipid metabolism is described mainly at oil plants, where they are commonly associated to storage and membrane composition, although these molecules could be identified at plants in general [39], [40]. Despite the high importance given to the studies about the lipids role at crop plants, the membrane lipid composition guarantees for plants the ability of persist under temperature stress, becoming essential for these organisms survival [39]. Teoh and co-workers [41] showed that 1.7% of total genes identified at maize embryos transcriptome were related to lipid metabolism, specially involved at fatty acid synthesis and fatty acid elongation. They found three genes related to lipid transfer proteins (LTPs) as well, which corroborates with our *in silico* results that will be expound at the sections bellow. Additionally, using the KOBAS web server [42] we were able to compare statistically significative pathways generated by KEGG for *Z. aethiopica* and *Arabidopsis thaliana*, *Oryza sativa* and *Vitis vinifera*. The KOBAS results mainly indicate that transcripts involved at circadian rhythm were more representative at our sample than the others organisms analyzed. Circadian systems will be discussed in more details at the following section.

### Circadian systems transcriptional signaling networks

**Figure 2:**
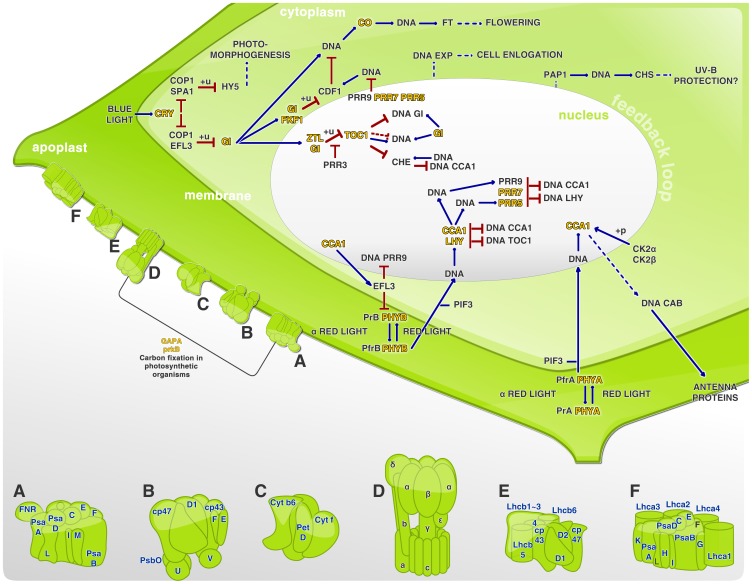
Circadian systems network at *Z. aethiopica* spathe. A. Photosystem I; B. Photosystem II; C. Citochrome complex b6/f; D. f-type ATPase; E. Light-harvesting chlorophyll complex II (LHCII); F. Light-harvesting chlorophyll complex I (LHCI); +u. ubiquitination; +p. phosphorylation; →. activation; —— direct effect; –. inhibition; ——. indirect effect. KOs found in this experiment was yellow highlighted, evidencing their role within each pathway. Intending to represent the photosynthesis process, photosystem I and II were showed as the transmembrane proteins structured on Figure 2A and 2B elements, even as the cytochrome b6/f complex demonstrated in 2C, and the light-harvesting chlorophyll protein complex in 2E and 2F, as LHCI and LHCII respectively. In order to underline the carbon fixation in photosynthetic organisms a line connecting the photosystem I (2A) and the ATPase (2D) was drawn, where it was possible to highlight the proteins phosphoribulokinase (prKB) and glyceraldehyde-3-phosphate dehydrogenase (NADP^+^) in the phosphorylating form (GAPA).

Involvement of circadian rhythmicity in such metabolic functions as photosynthesis, redox, energy generation, pH regulation and others has been extensively demonstrated, especially in higher plants. There are many indications that metabolism is not only an output of the circadian clock, but is intrinsically involved in its control [33], [43], [44]. Roenneberg and Merrow [43] have proposed that carbon fixation influences a decrease in CO_2_, which is dependent on photosynthesis rate, and that the photosynthesis rate is dependent on both CO_2_ concentration and pH. Also, Dodd et al. [45] demonstrated that the circadian system in *Arabidopsis* enhances the chlorophyll content, photosynthetic carbon fixation and growth. Additionally, the authors suggest that circadian enhancement of photosynthesis rates can improve plant survival, offering competitive advantages when comparison was made between the performance of wild-type plants and plants that presented mutations affecting control of the clock's period and length [45]. It was possible to discern 50 KOs related to photosynthesis, circadian rhythm and carbon fixation in the present study. These pathways are represented in Figure 2, which depicts a plant cell's essential compartments, allowing visualization of the above mentioned plant development and control pathways. Light control by photoreceptors, especially phytochromes and cryptochromes, can affect transcription and in some cases, directly affects the activity of transcription factors by influencing their post-translational modifications [46]. It was possible identify the genes CRY 1 and CRY 2 (cryptochrome), ZLT (ZEITELUPE), PFK1 (f-box related), CO (CONSTANS), LHY (late elongated hypocotyl transcription factor), TOC1 (timing of CAB expression 1), PRR7 (pseudo-response regulator 7) and PHYA (phytochrome A), that were specifically involved at the circadian rhythm control for plant environmental adaptation. Figure 2 shows the proteins involved in circadian rhythm starting with the blue light stimuli received by CRY. Also represented is the red light stimulus received by phytochrome A (PHYA) and phytochrome B (PHYB). These stimuli result in photoconversion of the proteins from the inactive form (Pr) to active form (Prf), and consequently lead to their translocation from the cytoplasm to nucleus [46]. There exists an integration point for light signals, and it may be suggested that the circadian clock associated 1 (CCA1) and LHY transcription factors constitute components of the plant circadian clock that provide a connection between light signals and the endogenous mechanisms triggered by their expression [44], [46]. It was demonstrated in *A. thaliana* mutant for *CCA1* and *TOC1* that misregulation of the circadian rhythm can cause a reduction in carbon fixation. This reduction results in a disadvantage for the mutants, since the incorrect matching of endogenous rhythms to environmental rhythms causes a reduction in chlorophyll content, assimilation and growth, and consequently increases mortality [45]. Studies with *A. thaliana* and rice demonstrated that CCA1 and LHY repress *TOC1*, while TOC1 closes the loop, positively controlling the expression of *CCA1* and *LHY*. In the same way GIGANTEA (GI) forms a feedback loop with CCA and LHY. Also the TOC1 paralogues pseudo-response regulator 5, 7 and 9 (PRR5, PRR7, PRR9) form a negative feedback looping with CCA and LHY [46]. Finally, ZTL controls TOC1 levels by designating it as a degradation target. Deeper studies are needed to further define these cross-talk processes between different parts of the clock [44], [46].

### Secondary metabolism role in *Z. aethiopica* defense and environmental response

Secondary metabolites are known for their role in a large number of plant metabolism and development processes such as energy production, growth and reproduction. Moreover, such compounds are capable of mediating in a fascinating way the plant's responses to abiotic and biotic environmental factors, acting for example as attractants for pollinators and seed dispersers, and also functioning as defensive compounds and toxins against pathogens and herbivores [47]. Owing to this remarkable action of secondary metabolites in plant defense, the KOs involved in biosynthesis and metabolism of secondary metabolites were evaluated, identifying 28 different pathways (Table 3), 13 of which may be involved in antimicrobial (bacterial and fungal) defense and six predicted to be related to herbivore defense. Also, seven pathways related to compound absorption and environmental detoxification were detected, as well as one involved in photooxidative stress, and others involved in floral physiological processes. Some of these functions could be grouped under more than one pathway description, such as antimicrobial activity and herbivore defense that appear to be integrated in almost all pathways. It was possible to map the secondary metabolite pathways which may be involved in antimicrobial activity, using the KEGG Orthology Database [48] and the KOs in each pathway were highlighted (Figures S4 to S17). These antimicrobial-related pathways were grouped into 11 biosynthesis pathways, which are: benzoxanoid (Figure S4), diterpenoid (Figure S5), flavone and flavonols (Figure S6), flavonoid (Figure S7), phenylpropanoid (Figure S8), polyketide sugar unit (Figure S9), sesquiterpenoid and triterpenoid (Figure S10), stilbenoid, diarylheptanoid and gingerol (Figure S11), streptomycin (Figure S12) and vancomycin (Figure S13) groups of antibiotics, tropane, piperidine and pyridine alkaloid (Figure S14) and two for degradation corresponding to naftalene (Figure S15) and polycyclic aromatic hydrocarbons (Figure S16). In each of these supplemental figures, the KOs obtained in this study are highlighted in red. Similar results were described for bamboo (*D. latiflorus*) floral transcriptome analysis, where 21 secondary metabolism subcategories were described, most of them being related to antimicrobial and herbivore defenses, followed by photooxidative stress and production of scent, essential oils and morphological differentiation [29]. Secondary metabolite biosynthesis was richly reported in the *C. tinctorius* transcriptome, where flavonoids, flavone and flavonols, isoquinoline alkaloids, tropane, piperidine and pyridine, glucosinolate, anthocyanin and betalaine biosynthesis were the most relevant pathways. Since plants often can produce secondary metabolites for defense, it is expected that a considerable number of genes related to secondary metabolism and biosynthesis be mapped to plant-pathogen interaction and hormonal signaling [32]. A painted spiral ginger (*Costus pictus*) transcriptome study also revealed a large number of transcripts involved in secondary metabolism, indicating the strong complexity of species, which is expected since it is well known that plants present around 25% of their genomes as specifying pathways related to natural product biosynthesis [49].

**Table 3:** Secondary Metabolism Pathways for *Zantedeschia aethiopica* assigned by KEGG Orthology (KO).

Pathway	KO Hits	Role of compounds in Plant	KO Codes
Terpenoid backbone biosynthesis	23	Herbivore defense	K00021, K00099, K00787, K00806, K00919, K00938, K00991, K01597, K01662, K01770, K01823, K03526, K03527, K05356, K05906K05954, K05955, K06013, K08658, K11778, K12742, K13789, K14066
Carotenoid biosynthesis	15	Photooxidative stress	K00514, K02291, K02293, K02294, K06443, K06444, K09835, K09837, K09838, K09839, K09840, K09841, K09842, K09843, K14606
Flavonoid biosynthesis	11	Herbivore and antimicrobial defense	K00475, K00660, K01859, K05277, K05278, K05280, K08695, K09754, K13065, K13082, K13083
Phenylpropanoid biosynthesis	7	Antimicrobial defense	K00083, K09753, K09754, K09755, K09756, K12355, K13065
Diterpenoid biosynthesis	7	Herbivore and antimicrobial defense	K04120, K04121, K04122, K04123, K04125, K05282, K13070
Zeatin biosynthesis	5	Cell division, flowering and chloroplast development	K00279, K00791, K10717, K10760, K13495
Brassinosteroid biosynthesis	5	Cell elongation and division, vascular differentiation, flowering, pollen development and photomorphogenesis	K09587, K09588, K09590, K12639, K12640
Benzoxazinoid biosynthesis	5	Allelopathy, antimicrobial and herbivore defense	K13223, K13227, K13228, K13229, K13230
Stilbenoid, diarylheptanoid and gingerol biosynthesis	3	Antimicrobial defense	K00517, K09754, K13065
Flavone and flavonol biosynthesis	3	Floral pigmentation, UV filtration, symbiotic nitrogen fixation, cell cycle inhibitor, antimicrobial defense	K05279, K05280, K13083
Atrazine degradation	2	Compound absorption and environmental detoxification	K01500, K03382
Aminobenzoate degradation	2	Compound absorption and environmental detoxification	K00517, K01101
Bisphenol degradation	2	Cell division and elongation, shoot differentiation	K00517, K05915
Indole alkaloid biosynthesis	2	Herbivore defense	K01757, K08233
Sesquiterpenoid and triterpenoid biosynthesis	2	Antimicrobial defense, pigmentation, ascent	K00511, K00801
Toluene degradation	1	Abiotic stress	K01061
Limonene and pinene degradation	1	Scent, morphological differentiation, essential oil component	K00517
Clorocyclohexane and clorobenzene degradation	1	Compound absorption and environmental detoxification	K01061
Streptomycin biosynthesis	1	Antimicrobial defense	K01710
Tropane, piperidine and pyridine alkaloid biosynthesis	1	Antimicrobial defense	K08081
Naphthalene degradation	1	Herbivore, nematode and antimicrobial defense	K05915
Fluorobenzoate degradation	1	Compound absorption and environmental detoxification	K01061
Polycyclic aromatic hydrocarbon degradation	1	Antimicrobial defense	K00517
Xylene degradation	1	Compound absorption and environmental detoxification	K10702
Aromatic compounds degradation	1	Compound absorption and environmental detoxification	K10702
Vancomycin group of antibiotics biosynthesis	1	Antimicrobial defense	K01710
Polyketide sugar unit biosynthesis	1	Antimicrobial defense, cell wall structure	K01710
Ethylbenzene degradation	1	Compound absorption and environmental detoxification	K10702

### Hormonal signaling network and defense role in *Z. aethiopica*

**Figure 3:**
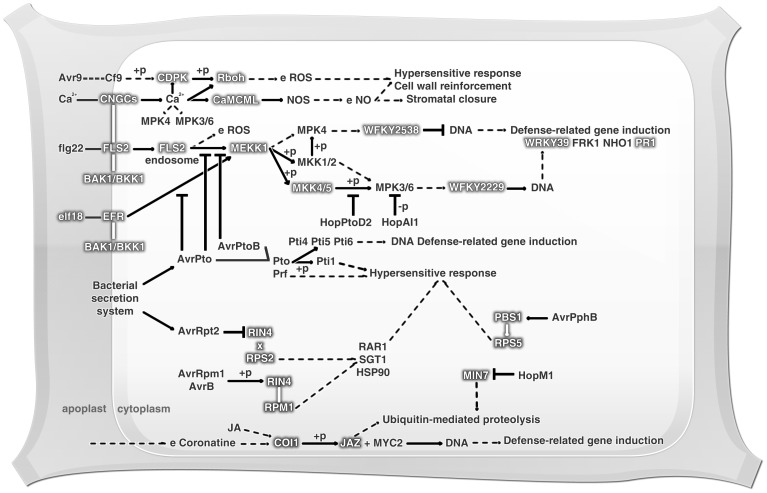
Hormonal signaling network at *Z. aethiopica* spathe. +u. ubiquitination; +p. phosphorylation; x. dissociation; →. activation; ——. direct effect; –. inhibition. Molecules identified in this study are highlighted with grey shadow.

**Figure 4:**
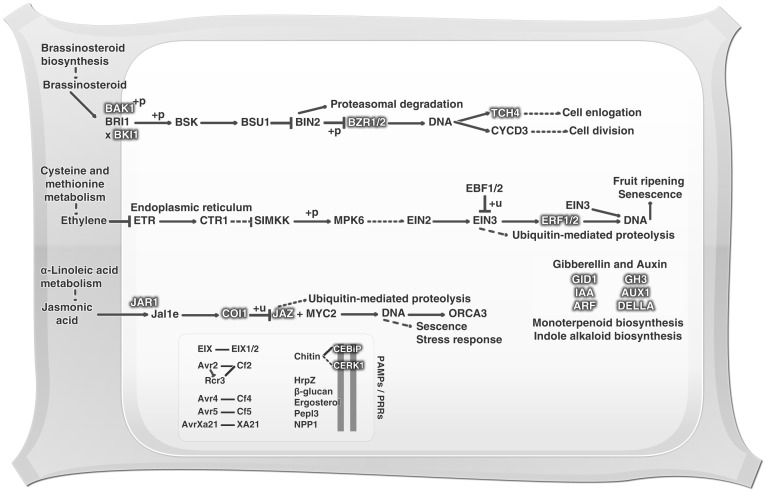
Plant-pathogen interaction basal immunity expression at *Z. aethiopica* spathe. e. expression; +u. ubiquitination; +p. phosphorylation; -p. dephosphorilation; x. dissociation; →. activation; ——. direct effect; –. inhibition. Molecules identified in this study are highlighted with grey shadow.

Plants can use constitutive defenses against predators and pathogens. In addition, they can employ inducible defense mechanisms, increasing the defensive capacity in parts of the plant distant form the area being attacked. The list of most effective molecules in defense pathways includes ethylene (ET), jasmonic acid (JA) and salicylic acid (SA) [50]. With the objective of drawing a floral defense profile, an investigation of basal defensive expression was performed, in which were identified 42 KOs related to plant hormone signaling, featuring the ET, JA, auxin (AU), gibberellin (GB) and brassinosteroid (BR) pathways, as shown in Figure 3. Also observed amongst the *Z. aethiopica* transcripts were the ethylene-responsive transcription factors (*ERF*) 1 and 2, and these are highlighted in the ethylene pathway in Figure 3. Ethylene is a gaseous plant hormone that is intrinsically related to germination, leaf and flower senescence, fruit ripening, and leaf abscission among other development processes. Moreover this hormone has been described as being involved in abiotic stress and pathogen attack response [51]. Furthermore, the *ERF* regulator has been extensively studied, mainly in the model plant *Arabidopsis*, and it is known as a *trans*-acting factor that binds specifically to GCC-box *cis*-acting elements in the promoter regions of ethylene-responsive genes [51]. ERFs can be controlled by conditions of cold, drought, pathogen infection, wounding or treatment with ET, SA or JA, and conversely, can regulate SA and JA-responsive genes [1], [51]. Pirrello et al. [51] suggested that these ERFs have a nuclear localization, but the mechanism of their transport to the nucleus is still unclear. ERF 1 and 2 have been known by their constitutive plant defense and stress response roles, and these are characteristically unique to plants [1], [52]. *ERF1* was demonstrated to be an early-ethylene responsive gene, and EIN3 protein expression is essential and sufficient to initiate the *ERF1* activation process. Moreover, ERF1 can promote *EIN3* expression [52], and ERF2 can also act as a transcriptional activator of GCC-box genes in the tobacco genome [53]. As with the ET pathway, it was possible to identify transcripts involved in the JA pathway, such as jasmonate ZIM-containing protein (*JAZ*), jasmonic acid-amino synthetase (*JAR1*) and coronatine-insensitive protein (*COI-1*). Recently the modes of action of COI-1 and JAZ have been elucidated. It is already known that in the presence of JA, JAZ proteins are targeted and degraded by the SCF/COI complex, which is an E3 ligase composed of Skp1, cullin, F-box protein and RING-H2 motif. This complex carries, in *Arabidopsis*, a positive JA signaling controller, specifically the MYC2/3/4 transcription factors and triggers JA-dependent gene expression [54]. JA can mediate processes including secondary growth, flower induction and defense response against biotic and abiotic stresses [54]. Furthermore, JA shows a conserved ability to elicit plant secondary metabolic pathways, as well as the flexible ability to communicate with other hormones such as GA, AU, ET, SA and abscisic acid (ABA). This property gives this hormone a multifaceted capacity to perform several roles in plant development and survival [55]. Four of these hormone-related KOs were identified as containing transcripts involved in the BR pathway, including the brassinosteroid insensitive 1-associated kinase 1 (*BAK1*), BRI1 kinase inhibitor 1 (*BKI1*), brassinazole resistant (*BZR*) *1* and *2* and touch gene 4 (*TCH4*) (Figure 4). The BRs are steroid hormones involved in diverse biological processes such as cell elongation, flowering, and resistance to biotic and abiotic stresses among others [56]. BAK1 is a receptor kinase (LRR-RK) required for a rapid association with FLS2 and EFR (EF-Tu receptor, positive regulator), where it promotes a integration between PAMP recognition and signaling response [57], [58]. BAK1 is also associated with brassinosteroid insensitive 1 (BRI1), which is a BR hormone receptor. In this case BAK1 acts as a positive regulator in the control of plant growth and development [58]–[60]. Moreover, the inhibitory plasma membrane phosphoprotein BKI1 interacts in a highly specific manner with the BRI1 kinase domain, influencing BRI1-BAK1 interaction, due to its requirement for the formation of the BRI1-BAK1 complex [60], [61]. These events lead to *BRI1* activation and phosphorylation of cytoplasmic BR signaling kinases (BSK), thereby inducing *BZR1* transcription [60]. *BZR1* expression can be negatively controlled by BR insensitive 2 (BIN2), resulting in dephosphorylation of the nuclear protein BZR1, thereby causing a slowing of plant development [56]. Furthermore, BRI1 appears as a positive regulator of *TCH4*. TCH4 acts as an endoxyloglucan transferase, and, when controlled by BR, AU or some abiotic stimuli, can participate in cell wall modifications [48], [62]. It has been observed that AU is a plant growth and development controller [35], and this hormone pathway was also represented in this study by several predicted genes including the auxin influx carrier (*AUX1*), auxin-responsive GH3 family (*GH3*), auxin response factor (*ARF*) and auxin-responsive protein indole-3-acetic acid (*IAA*). AU stimuli related to the genes *GH3* and *IAA* have been intensively studied, and these are considered the primary AU-responsive genes [41]. It is reported that the AUX and IAA proteins can negatively control *ARF*, acting as an *ARF* transcriptional repressor complex, and this complex (AUX/IAA) may also be involved in plant-pathogen interaction, for example by participating in recognition of the flagelin *flg22* elicitor by the plant [35], [40]. Additionally, the GH3 protein family can also be regulated by ARFs, but not all GH3 proteins are auxin-responsive. Arabidopsis [63] and rice [37], [64] plants that overexpress GH3 demonstrate an auxin-deficient phenotype, evidenced by basal plant disease resistance associated with auxin signaling suppression [35], [65]. Also, AU-induced genes are known for their action at the circadian clock by modulating the AU signaling, as at the plant growth, which can be regulated by exogenous AU stimuli [34]. Furthermore, the GA-insensitive dwarf 1 (GID1) was identified, which is a receptor that binds to GA and interacts with DELLA proteins (negative GA action regulators) [66], which were also identified here. GID1-GA interaction with DELLA proteins results in DELLA degradation by the SCF complex (as described above for JA), which triggers GA action. GA is a large family of tetracyclic diterpenoid hormones that is involved in seed germination, stem elongation, pollen maturation and transition from vegetative growth to flowering, besides coordinating responses to environmental stresses such as low temperature and osmotic stress [38], [66], [67]. The loss of response of one hormone cannot be compensated by other hormone responses. Thus the entire plant defense response can be altered by temperature and light modifications [61]. Notwithstanding, there is a clear communication between the complete plant hormone signaling network and the secondary metabolism system. These interconnections lead to a better understanding of the plant defense, particularly in terms of the basal defense capability expressed in the plant in the absence of a pathogen challenge [35].

### *Z. aethiopica* innate immunity against pathogens

Plants have two strategies for innate immune response against microbes. One of these strategies is the use of pattern recognition receptors (PRRs) on the external face of the host cell and illustrates the plant defense system's first step for pathogen perception. PRRs are responsible for pathogen-associated molecular patterns (PAMP) recognition and for activating PAMP-triggered immunity (PTI) [68]. The second perception step involves the recognition of pathogen virulence effectors by intracellular receptors. As a result of this recognition, effector-triggered immunity is induced (ETI) [59]. 22 KOs related to plant-pathogen interaction were found, and it is of interest to remark that 5 of them (K13422-*MYC2*; K13464-*JAZ*; K13416-*BAK1*; K13449-*PR1* and K13463-*COI1*) form a connection between hormone signaling and plant-pathogen interaction pathways. In this study PRRs involved in bacterial and fungal response signaling were observed. The flagelin-sensing 2 gene (*FLS2*), highlighted in Figure 4, is described as encoding a PRR leucine-rich repeat receptor kinase (LRR-RK) that is able to recognize bacteria by the conserved 22-amino-acid epitope, flg22, present in the flagellin N-terminus. Functional *FLS2* orthologous were demonstrated in *A. thaliana*, tobacco and rice [59], [69]. The absence of FLS2 flagelin perception in *Arabidopsis* and tobacco caused an enhanced susceptibility to virulent strains, showing that FLS2 is involved in bacterial resistance [70]-[73]. Another LRR-RK found in the *Z. aethiopica* transcriptome was the EF-Tu receptor (EFR) (Figure 4). This PRR recognizes the bacterial elongation factor Tu (EF-Tu) and other proteins containing the conserved elf18 peptide (a conserved sequence of 18 N-terminal residues). It was shown that *Arabidopsis* EFR mutants are more susceptible to *A. tumefaciens* [74] and hypo-virulent strains of *Pseudomonas syringae* [68]. Usually, PRRs are protein kinases responsible for recognizing extracellular signals and stimulating the intracellular signaling cascade for defense activation [75]. However, these proteins do not act alone, requiring other signaling proteins such as BAK1, described above for BR hormonal signaling, to complete the signaling cascade [57]. Transcripts encoding PRRs involved in fungal perception were also identified here. Among them the transmembrane protein CEBiP (chitin elicitor binding protein), with two extracellular LysM motifs and a short cytoplasmic tail was found (Figure 4). CEBiP is able to recognize the cell wall chitin of many superior fungi, and it is known that chitin is a potent PAMP in a wide variety of plants [68]. When *CEBiP* expression was silenced in rice there was a reduction in binding and responses triggered by chitin [59]. Another PRR able to recognize fungal chitin found in the transcriptome was the chitin elicitor receptor kinase (CERK1). CERK1 is a receptor involved in recognition of both fungi and bacteria. Arabidopsis plants with Ds-transposon insertion mutations in the gene *cerk1* are more susceptible to *Erysiphe cichoracearum* and *Alternaria brassicicola* fungi [76], [77]. Interestingly, the same *Arabidopsis* mutant plants were shown to be more susceptible to *P. synringae*, evidencing the role of CERK1 in bacterial and fungal resistance [57], [76]–[78]. Figure 4 shows the cellular events associated with both PTI and ETI responses. These involve a rapid influx of calcium ions from the external membrane, a burst of reactive oxygen species (ROS), activation of mitogen-activated protein kinases (MAPKs), reprogramming of gene expression through transcription factors (WRKY) and deposition of callosic cell wall. These events are known to occur in plants after contact with pathogens, often ending in a localized cell death hypersensitive response (HR) and the production of antimicrobial molecules [79].

### Antimicrobial Peptide Screening

**Figure 5:**
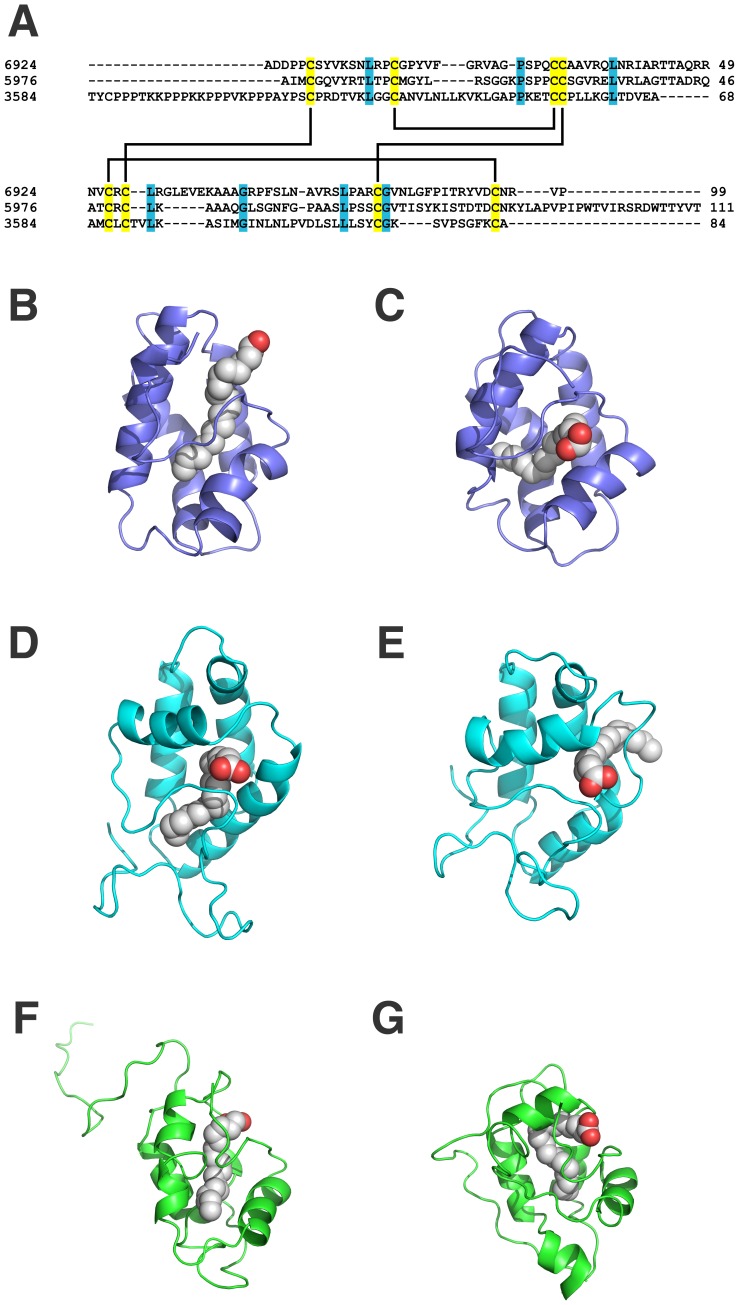
Lipid Transfer Proteins structural analysis. A. Multiple alignment of LTPs here identified. Conserved residues are green highlighted and the cysteine residues are in yellow. Final three-dimensional structures of LTPs with ligands before and after 50 ns of molecular dynamics are illustrated at B. Za-LTP1 + Oleic acid initial; C. Za-LTP1 + Oleic acid final; D. Za-LTP2 + Palmitoleic acid initial; E. Za-LTP2 + Palmitoleic acid final; F. Za-LTP3 + Alpha-Linoleic acid intial and G. Za-LTP3 + Alpha-linoleic acid final.

The initial detection of antibacterial activity in the *Z. aethiopica* spathe protein-rich extracts provided enough evidence to instigate a search for AMPs within the transcriptome data set. Almost all plant AMP families are cysteine-rich and have a globular structure stabilized by dissulphide bonds (S-S). In floral organs, defensins, LTPs, myrosinase-binding proteins, heveins, cyclotides and snakins have been described [3], [80], [81]. In the current study, it was possible to identify, through data mining analysis, three of these classes of flower AMPs. An *in silico* screening was carried out for AMP detection. Using the cysteine-rich AMP patterns proposed by Silverstein et al. [82] (Table S1), Perl scripts were applied to search for peptides containing such patterns while avoiding sequences longer than 350 amino acid residues, thereby guaranteeing antimicrobial peptide sequence selection. Following the established parameters it was possible to distinguish 34 sequences containing the above-mentioned AMP patterns. From these sequences, 15 showed a signal peptide and no transmembrane domains after Phobius analysis, predicting the possibility of secretion by the cell. However, just 10 sequences showed an AMP domain, according to InterPro Scan. Using this tool it was possible to identify six lipid transfer proteins (LTP), three snakin/GASA proteins and one chimerolectin containing a hevein domain as potential AMPs. Finally, only three potential LTPs presented positive prediction by CS-AMPPred, here identified as Za-LTP1, Za-LTP2 and Za-LTP3. However, the cysteine-rich patterns proposed by Silverstein et al. [82] for LTPs are not very restrictive, and embrace 2S albumins and ECA1 classes. For this reason, structural models were constructed to confirm LTP characterization. The molecular models indicated that the three predicted LTPs have a typical LTP fold, with a four-helix bundle, stabilized by four disulfide bonds (the validation parameters are summarized in Table S2). A search was performed to verify the capacity of these LTPs to interact with lipids. Using the COFACTOR server one different lipid was predicted to interact with each LTP model (Table 4). Figure 5 illustrates the LTPs and their respective ligands: Za-LTP1 and linoleic acid (OLA) (Figure 5B and 5C), Za-LTP2 and palmitoleic acid (PAM) (Figure 5D and 5E), and Za-LTP3 and alpha-linoleic acid (LNL) (Figure 5F and 5G). A multiple sequence alignment (Figure 5A) shows a conserved cysteine pattern for the three LTPs studied here. The stability of the complexes was evaluated through molecular dynamics, where the affinity between the LTPs and the lipid molecules was observed during 50 ns of simulation. RMSD and TM-Score (Table 4) indicated that structural folding of the LTPs was maintained. The data mining method here applied suggests three reliable AMP candidates (Za-LTP1, Za-LTP2 and Za-LTP3) in the *Z. aethiopica* transcriptome through *in silico* experiments. The LTPs are expressed mainly in epidermal cells of leaves and flowers [83], and were involved in many biological processes such as cutin biosynthesis, somatic embryogenesis, anther development and defense [84]. LTPs have been largely studied in relation to their antimicrobial potential, such as the non-specific LTPs from barley, spinach and *Arabidopsis* leaves that are able to inhibit the growth of the bacteria *Clavibacter michiganensis*, *Pseudomonas solanacearum* and the fungus *Fusarium solani* [85], [86]. Notwithstanding, *in vitro* studies remain necessary to verify the antimicrobial activity of the LTPs identified in the present study.

**Table 4:** Final molecular dynamics scores for *Zantedeschia aethiopica* lipid transfer protein (LTP) docking with lipid ligands.

LTP	COFACTOR			Molecular Dynamics^a^	
	Ligand	BS-Score	PDB Hit	RMSD (Å)^b^	TM-Score
Za-LTP1	Linoleic Acid (OLA)	1.54	1FK5	3.16	0.69
Za-LTP2	Palmitoleic Acid (PAM)	1.62	1UVB	3.55	0.68
Za-LTP3	Alpha-Linoleic Acid (LNL)	1.01	1FK6	10.79^c^	0.50

a Data generated by comparing the structure at 0 ns and 50 ns.

b The RMSD evolution along the time is available in Figure S14.

c Excluding the polyproline tail, this value is reduced to 4.117.

## Conclusions

The sequence data provided by RNA-Seq technology proved to be useful in characterizing the transcriptome of the non-model plant *Z. aethiopica*. Through the use of functional profile construction, we are able to verify the existence of a high level of plant spathe investment in employing genetic information and metabolism-related transcription processes. Moreover, carbohydrate, amino acid and secondary metabolites-related transcription were demonstrated to be prioritized. These events may indicate an investment in organism homeostasis maintenance, since there was a clear interaction between the circadian, hormonal signaling and basal immunity systems. This communication among systems affords an impressive regulatory potential for triggering multiple resistance mechanisms and could support the plant in arranging the activation of one specific system over another, thereby maintaining an optimal defense against pathogens or predators. Furthermore, the transcriptome analysis of *Z. aethiopica* offers valuable information about this plant, providing new insights regarding the plant's physiology and molecular mechanisms. Ultimately, the antibacterial activity observed for *Z. aethiopica* spathe can be associated with a high content of secondary metabolites as described in the functional profile and, additionally, the LTPs identified here can play a crucial role in plant primary defense.

## Materials and Methods

### Prospection of floral tissues with antimicrobial properties

#### Flower material selection and protein-rich fraction extraction

For antimicrobial compound prospection from floral tissues, a selection of 10 ornamental and Cerrado plants was performed. From all studied vegetal species, 50 units of each floral structure were used for protein-rich fraction acquisition (Table 1). In order to obtain the protein rich fraction, an acid extraction (HCl 1% with 0.6 M NaCl) in a 1∶3 proportion (w/v) was carried out. The suspension was centrifuged at 8000×g, for 30 min, at 4°C. The supernatant was subsequently precipitated using a 100% (w/v) concentration of ammonium sulfate, centrifuged at 8000×g, at 4°C, for 30 minutes and extensively dialyzed against distilled water (cutoff of 500 Da). Posteriorly, the samples were lyophilized and re-suspended in sterile distilled water. Protein quantification was performed using the RC DC Protein Assay (Bio-Rad, http://www.bio-rad.com/) according the manufacturer's recommendations.

#### Antibacterial bioassays

The antibacterial activity evaluation was conducted by microspectrophotometry using 96-well microplates according to the recommendations of the National Committee for Clinical Laboratory Standards [87] with minor modifications. The analyses against *Escherichia coli* (ATCC 8739) and *Staphylococcus aureus* (ATCC 29213) were performed in LB broth (Luria-Bertani, pH 7.0). Initially, a growth curve of original culture was established in order to determine the relationship between colony forming units (CFU) and optical density at 595 nm. For antibacterial activity evaluation, 0.1 mL of inoculum was cultured in 4.0 mL of LB medium for 3 h, at 37°C, until the mid-logarithmic-phase was obtained. After this incubation time, an aliquot corresponding to 5×10^5^ CFU mL^−1^ and 200 µg mL^−1^ of total protein was added to a solution of LB broth, to a final volume of 0.1 mL in the microplate wells. Microplates were incubated at 37°C and bacterial growth was monitored at 595 nm, every 30 min, until it achieved a stationary phase. The growth inhibition was measured by spectrophotometry (595 nm) and the inhibition percentage was calculated considering the positive control values as 100% of inhibition and negative control was defined as 0% of inhibition. Chloramphenicol at a concentration of 60 µg mL^−1^ was used as a positive control. Distilled sterile water was used as a negative control. All bioassays were performed in triplicate.

### Transcriptome Analysis

#### Sample preparation and RNA extraction

*Z. aethiopica* spathe tissue (50 individuals) was used for the transcriptome analysis. The spathes were acquired from local market, in adult growth stage and free from abiotic or biotic stress stimuli. One gram of tissue was ground in liquid nitrogen using an autoclaved ceramic mortar and pestle, and total RNA was then extracted using the InviTrap Spin Plant RNA Mini Kit (Invitek, http://www.invitek.de/), according the manufacturer's recommendations in order to guarantee the RNA integrity. RNA concentration was determined using the Qubit RNA Assay Kit (Invitrogen, http://products.invitrogen.com) and integrity checked by unidimensional electrophoresis in an agarose gel [88]. The total RNA eluted was precipitated using ethanol until RNA library construction.

#### Library construction and RNA-Seq

RNA library construction for the *Z. aethiopica* sample and sequencing was done at the Tufts University Core Facility. The RNA library was prepared using Illumina's TruSeq RNA preparation kit following manufacturer's instructions. Sequencing was carried out for 100 cycles (paired-end reads) on one lane of an Illumina HiSeq 2000 platform. Sequence data from this article can be found in the EMBL/GenBank data libraries under accession number of Sequence Read Archive: SRR868662.

#### Data pre-processing and transcriptome assembly

Raw Illumina sequence data were pre-processed using the Trimmomatic tool to eliminate sequencing adaptors using a customized fasta file containing all known Illumina adaptors, and also to remove low-quality sequence reads by scanning the read with a 5-base sliding window, cutting when the average quality per base dropped below 15 [27]. The resulting sequence reads below 36 bp long were excluded. *De novo* transcriptome assembly was carried out with Trinity assembler (Meryl k-mer counter) [28], using both paired-end and single-end high quality reads as inputs. The processed transcriptome assembly was deposited as a Transcriptome Shotgun Assembly project at DDBJ/EMBL/GenBank under the accession GAOT00000000. The version described in this paper is the first version, GAOT01000000.

#### Functional Profile

Gene names were assigned using the GeneMark software [89], using *Arabidopsis thaliana* as a model [90]. Moreover, genes were also predicted by the TransDecoder tool [30] using the default parameters. All reads were remapped using the set of all transcripts assembled by Trinity [30] as reference. For mapping the Bowtie2 tool was used [91], applying the default parameters. The mapping coverage was performed using the tool coverage Bed from BedTools package. This tool shows how many reads were mapped within a certain region, which cover the same region. Regions were considered for these analyses were the coding sequences predicted by the TransDecoder and its boundaries were reported in the GFF file generated during the prediction. The comparison of GeneMark and TransDecoder tools was demonstrated by using a Venn diagram (Figure S2). Gene coverage was also verified during the remapping as demonstrated on Figure S3 and Table S3. Unigene functional profiling was performed by alignment to the plant non-redundant proteins database from the National Center for Biotechnology Information (NCBI NR, http://www.ncbi.nlm.nih.gov/) and The Kyoto Encyclopedia of Genes and Genomes (KEGG, http://www.genome.jp/kegg/) [92], based on *Arabidopsis* annotation, using BLASTX (significance threshold: e-value <1.0e^−5^). The pathway assignments were mapped according the KEGG database. Also was submitted the KEGG data to the KOBAS web server [42] analysis for identification and annotation of enriched pathways, using as comparison organisms *A. thaliana*, *O. sativa* and *V. vinifera*.

### Antimicrobial Peptide Screening

In order to identify antimicrobial peptide transcripts in the *Z. aethiopica* data set the cysteine-rich antimicrobial peptide pattern proposed by Silverstein et al. [82] was used to describe the classes LTP/2S albumin/ECA 1, defensin, hevein, thionin and snakin/GASA/GAST (Table S1), through Perl scripts. The script was adjusted to select the sequences containing antimicrobial patterns, restricting the maximum size to 350 amino acid residues, as described by Porto et al. [93]. The selected transcripts were further submitted to Phobius analysis [94] for signal peptide and transmembrane region identification. Subsequently the sequences without signal peptides and the sequences with transmembrane domains were discarded. The remaining sequences were then submitted to InterProScan [95] for domain identification, where the largest domain signature was chosen as the actual domain. Thereafter sequences with tails longer than 30 amino acid residues were removed. Subsequently all the remaining sequences were submitted to CS-AMPPred [96], [97] for antimicrobial activity prediction, where the sequences positively predicted in two or three CS-AMPPred models were selected. The software CS-AMPPred is a model of support vector machine (SVM) designed specifically to predict cysteine-stabilized antimicrobial peptides. Figure 5 shows a multiple alignment constructed by ClustalW [42] using the sequences predicted as antimicrobial by CS-AMPPred.

#### Molecular Modeling

The LOMETS server [98] was used to find the best template for comparative modeling. LOMETS is a meta-threading server which collects the information from nine threading servers and then ranks that information regarding the templates. The best template was selected taking into account the coverage and identity from the alignments. As such, one hundred theoretical three-dimensional models were constructed through Modeller 9.10 [99]. The models were constructed using the default methods of the automodel class. The final models were selected according to the discrete optimized protein energy (DOPE) scores. This score assesses the energy of the model and indicates the best probable structures. The model with the best DOPE score was evaluated through PROSA II [100] and PROCHECK [101]. PROCHECK checks the stereochemical quality of a protein structure, through the Ramachandran plot, where reliable models are expected to have more than 90% of the amino acid residues in the most favored and allowed regions, while PROSA II indicates the fold quality. Structure visualization was done in PyMOL (The PyMOL Molecular Graphics System, Version 1.4.1, Schrödinger, LLC, http://www.pymol.org).

#### Structural Alignments and Ligand Predictions

Structural alignments were performed in two ways, first through the Dali server [102], where the assessment of structural alignments was done through the Z-score, a structural alignment with Z-score higher than 2 being significant. In addition, the COFACTOR server [103] was used as a second method. COFACTOR uses the TM-align structure alignment program [104] to search against the PDB and then examines the binding pockets, predicts the binding pose of ligands in the target structure or model and constructs the protein-ligand complexes. Therefore, this approach allows the identification of the binding position of ligands without docking experiments.

#### Molecular Dynamics

Molecular dynamic simulations (MD) of the protein-ligand complexes were performed in a water environment, using the Single Point Charge water model [105]. The analyses were carried out by using the GROMOS96 43A1 force field and computational package GROMACS 4 [106]. The dynamics used the three-dimensional models of the protein-ligand complexes as initial structures, immersed in water molecules in cubic boxes with a minimum distance of 0.7 nm between the complexes and the boxes frontiers. Chlorine ions were also inserted into the complexes with positive charges in order to neutralize the system charge. Geometry of water molecules was constrained by using the SETTLE algorithm [107]. All atom bond lengths were linked by using the LINCS algorithm [108]. Electrostatic corrections were made by Particle Mesh Ewald algorithm [106], with a *cut off* radius of 1.4 nm in order to reduce the computational time. The same *cut off* radius was also used for van der Waals interactions. The list of neighbors of each atom was updated every 10 simulation steps of 2 fs. The conjugate gradient and the steepest descent algorithms (50,000 steps each) were implemented for energy minimization. After that, the system temperature was normalized to 300 K for 2 ns, using the Berendsen thermostat (NVT ensemble). Additionally the system pressure was normalized to 1 bar for 2 ns, using the Berendsen barostat (NPT ensemble). The systems with minimized energy, and balanced temperature and pressure were simulated for 50 ns by using the leap-frog algorithm. The simulations were evaluated through RMSD and DSSP. Final scores values were organized at Table 4.

## Supporting Information

Figure S1 **Evaluation of RNA extraction quality of** ***Zanthedeschia aethiopica*** **sample.** RNA was resolved at 1% agarose-gel electrophoresis in 100 mM Tris buffer. The gel was subsequently stained with 0.5 mg/mL of Ethidium bromide. MM: Molecular marker; Z.A.: *Z. aethiopica* RNA sample.
(TIFF)

Figure S2 **Venn diagram comparison of GeneMark and TransDecoder tools gene prediction.**
(TIFF)

Figure S3 **Gene coverage histogram of remapping through TransDecoder tool.**
(TIFF)

Figure S4 **Benzoxanoid biosynthesis.**
(TIFF)

Figure S5 **Diterpenoid biosynthesis.**
(TIFF)

Figure S6 **Flavone and flavonol biosynthesis.**
(TIFF)

Figure S7 **Flavonoids biosynthesis.**
(TIFF)

Figure S8 **Phenylpropanoid biosynthesis.**
(TIFF)

Figure S9 **Polyketide sugar unit biosynthesis.**
(TIFF)

Figure S10 **Sesquiterpenoid and triterpenoid biosynthesis.**
(TIFF)

Figure S11 **Stilbenoid, diarylheptanoid and gingerol biosynthesis.**
(TIFF)

Figure S12 **Streptomycin biosynthesis.**
(TIFF)

Figure S13 **Biosynthesis of vancomycin antibiotics group.**
(TIFF)

Figure S14 **Tropane, piperidine and pyridine alkaloid biosynthesis.**
(TIFF)

Figure S15 **Naftalene degradation.**
(TIFF)

Figure S16 **Polycyclic aromatic hydrocarbon degradation.**
(TIFF)

Figure S17 **RMSD backbones of lipid-transfer proteins during 50 ns of molecular dynamics.** A. Za-LTP1; B. Za-LTP2; C. Za-LTP3.
(TIFF)

Table S1 **Cysteine-rich antimicrobial peptides pattern.**
(PDF)

Table S2 **Summary of validation parameters for structural three-dimensional models.**
(PDF)

Table S3 **TransDecoder remapping analysis.**
(PDF)
