# Supplementary figures and images for: Shedding Some Light over the Floral Metabolism by Arum Lily (Zantedeschia aethiopica) Spathe De Novo Transcriptome Assembly
Source: PLoS One. 2014 Mar 10;9(3):e90487. doi: 10.1371/journal.pone.0090487 (PMC3948674; doi:10.1371/journal.pone.0090487)

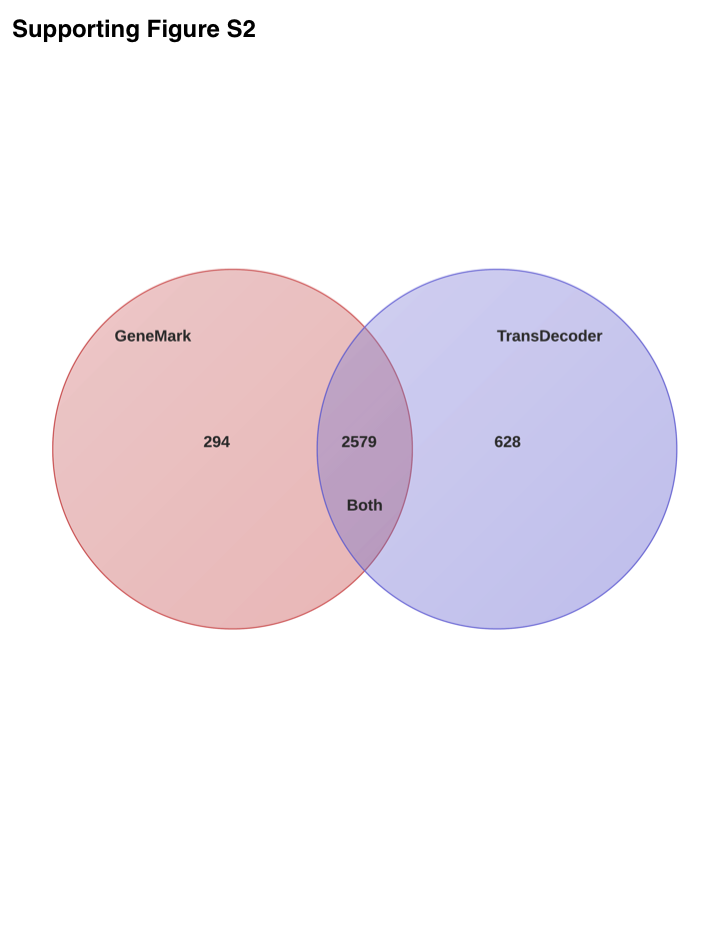

Supplement: Figure S2 — Venn diagram comparison of GeneMark and TransDecoder tools gene prediction. (TIFF) [file pone.0090487.s002.tiff]

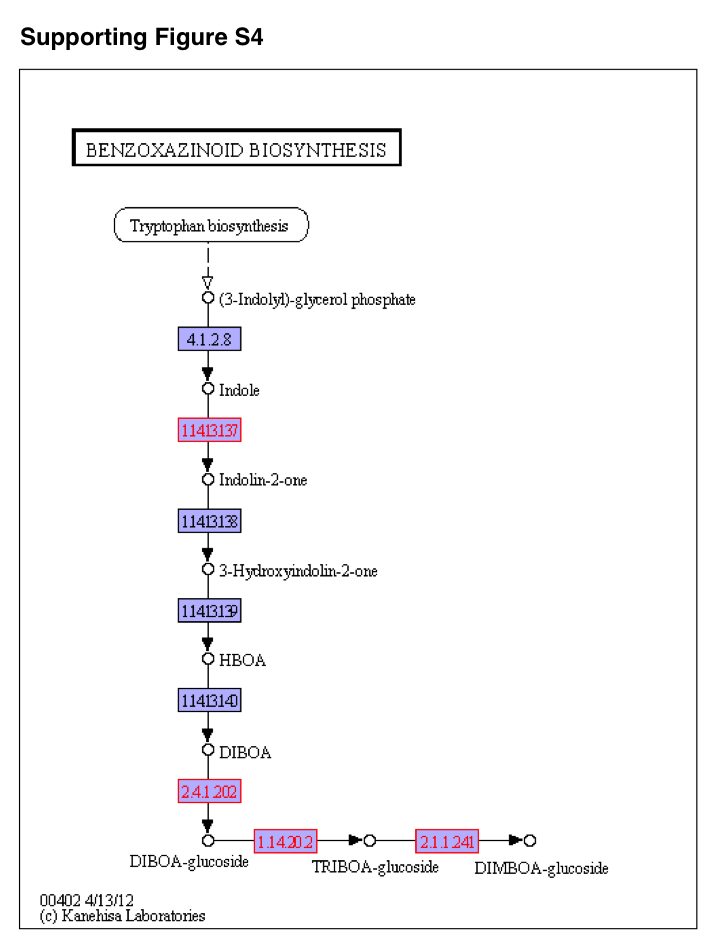

Supplement: Figure S4 — Benzoxanoid biosynthesis. (TIFF) [file pone.0090487.s004.tiff]

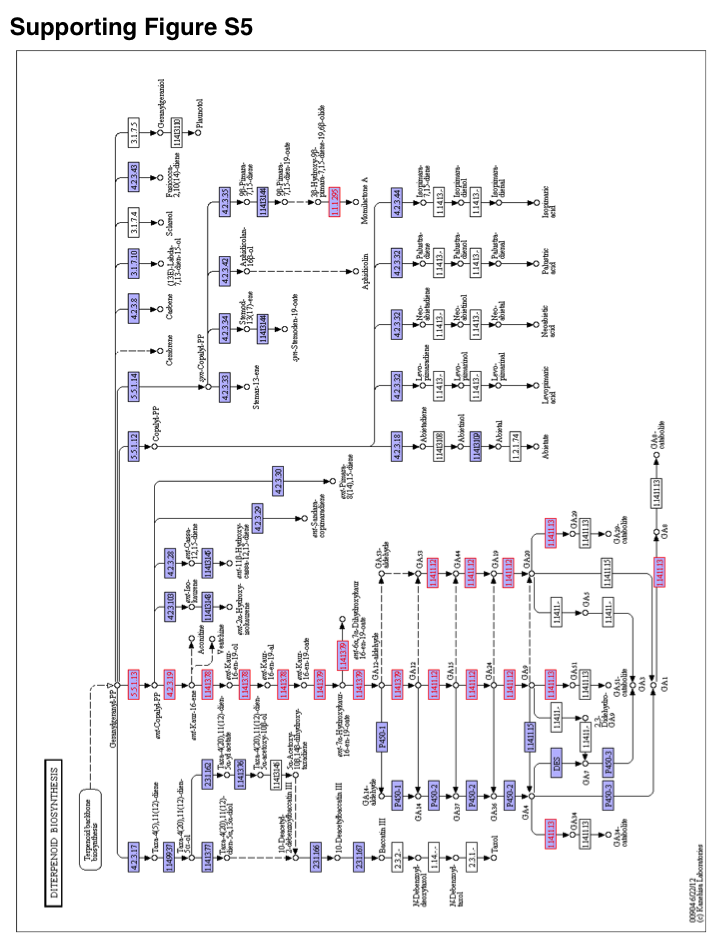

Supplement: Figure S5 — Diterpenoid biosynthesis. (TIFF) [file pone.0090487.s005.tiff]

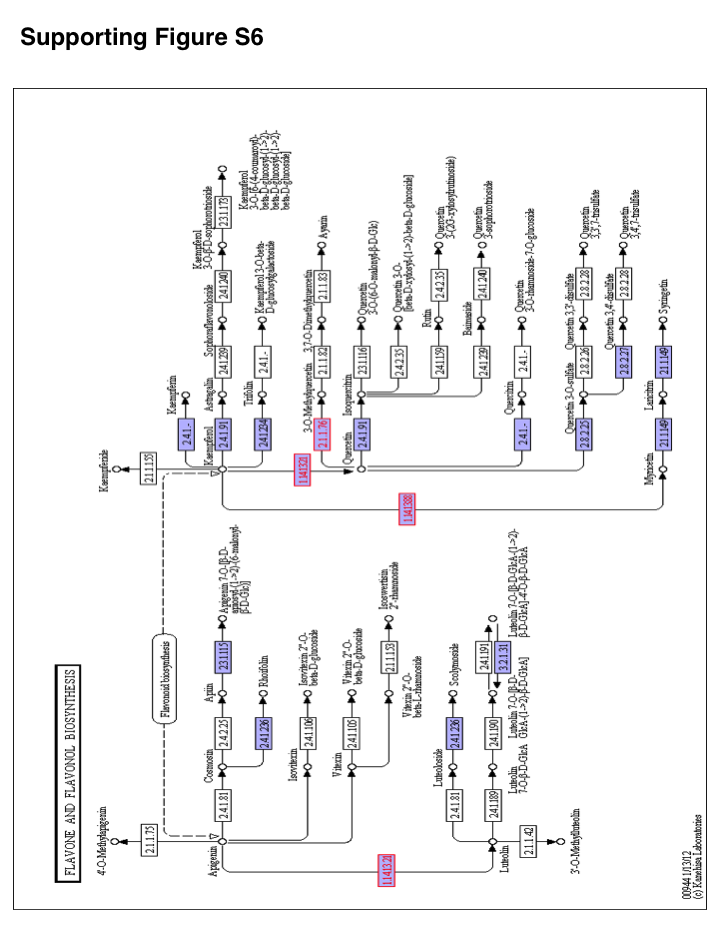

Supplement: Figure S6 — Flavone and flavonol biosynthesis. (TIFF) [file pone.0090487.s006.tiff]

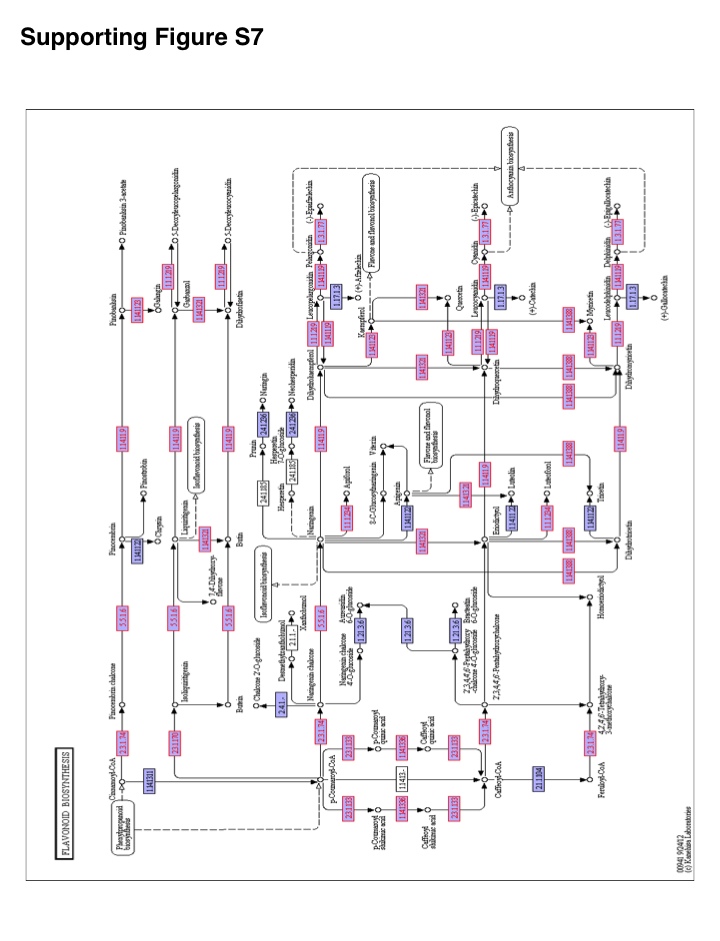

Supplement: Figure S7 — Flavonoids biosynthesis. (TIFF) [file pone.0090487.s007.tiff]

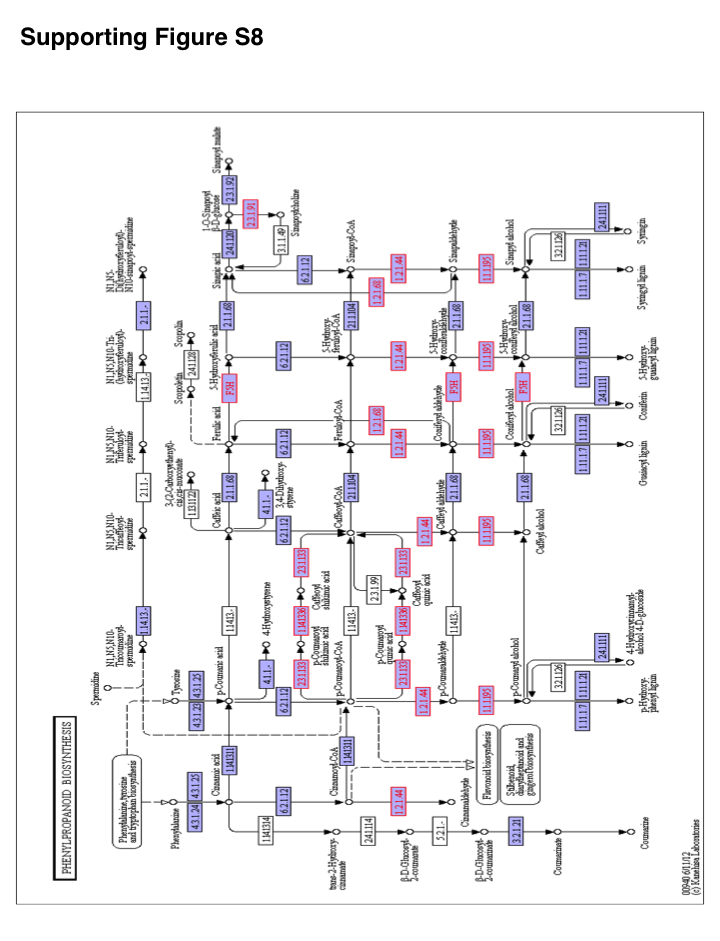

Supplement: Figure S8 — Phenylpropanoid biosynthesis. (TIFF) [file pone.0090487.s008.tiff]

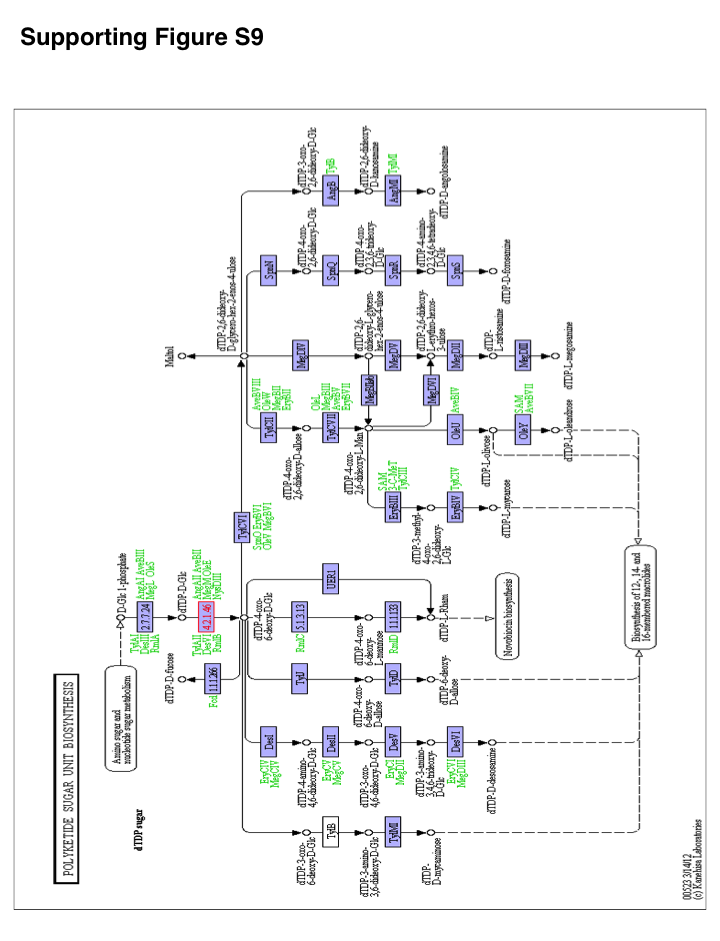

Supplement: Figure S9 — Polyketide sugar unit biosynthesis. (TIFF) [file pone.0090487.s009.tiff]

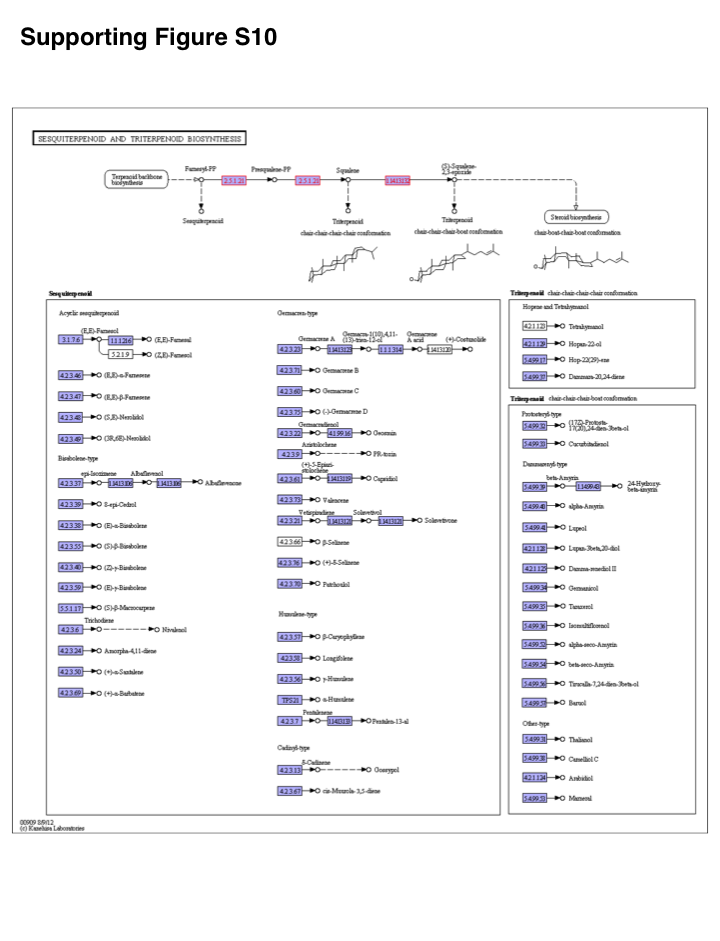

Supplement: Figure S10 — Sesquiterpenoid and triterpenoid biosynthesis. (TIFF) [file pone.0090487.s010.tiff]

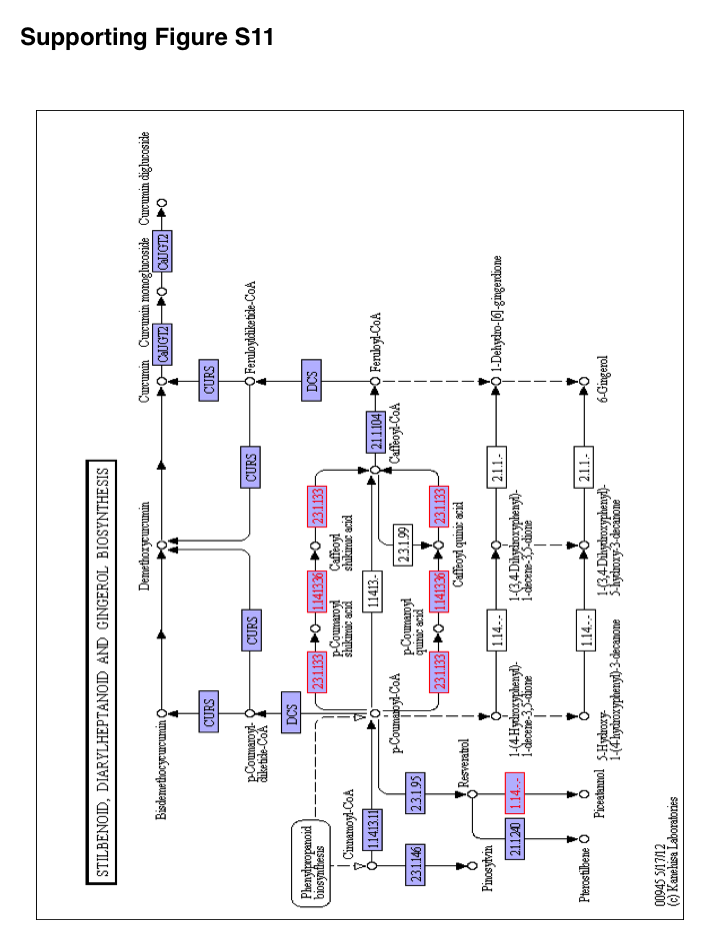

Supplement: Figure S11 — Stilbenoid, diarylheptanoid and gingerol biosynthesis. (TIFF) [file pone.0090487.s011.tiff]

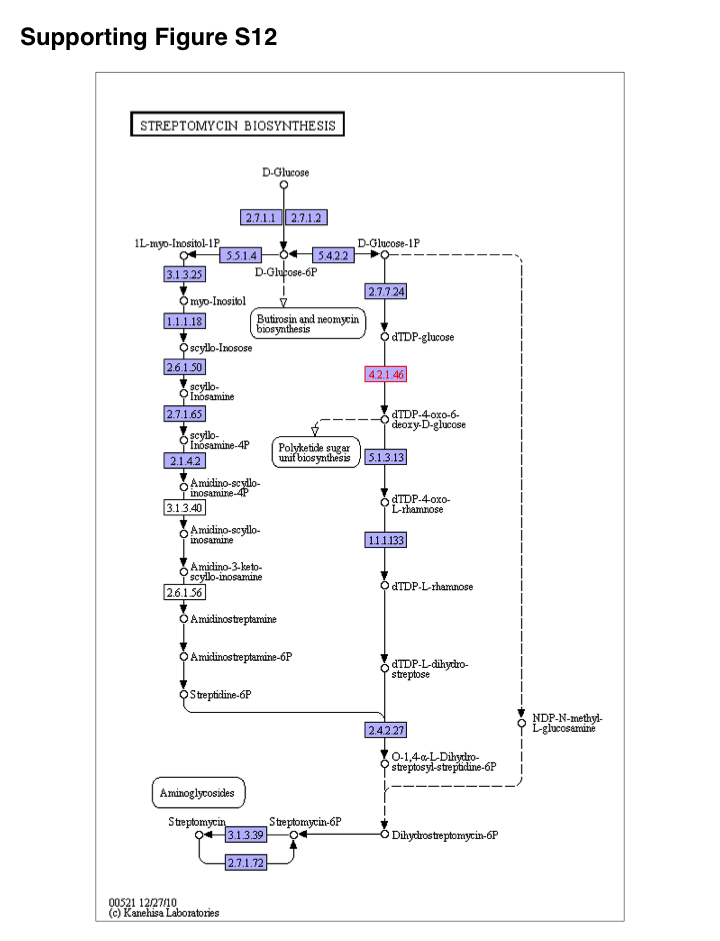

Supplement: Figure S12 — Streptomycin biosynthesis. (TIFF) [file pone.0090487.s012.tiff]

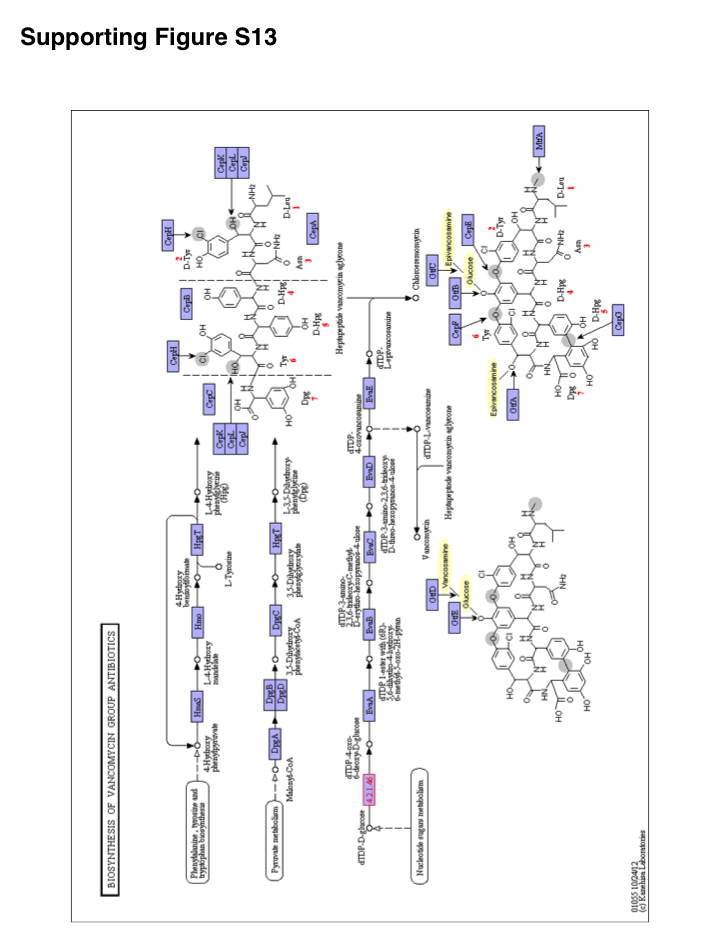

Supplement: Figure S13 — Biosynthesis of vancomycin antibiotics group. (TIFF) [file pone.0090487.s013.tiff]

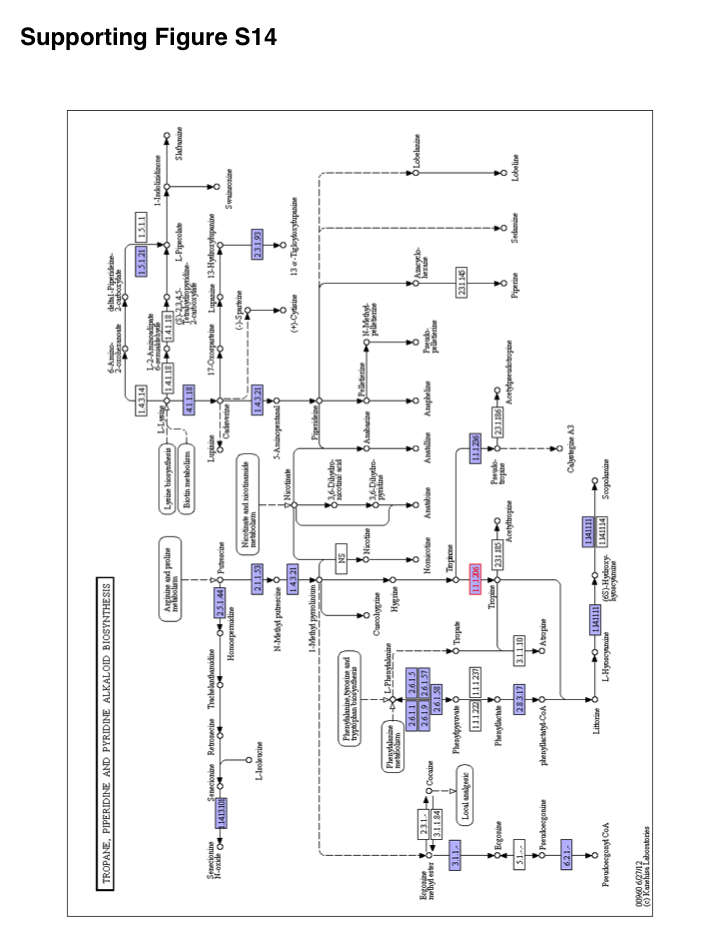

Supplement: Figure S14 — Tropane, piperidine and pyridine alkaloid biosynthesis. (TIFF) [file pone.0090487.s014.tiff]

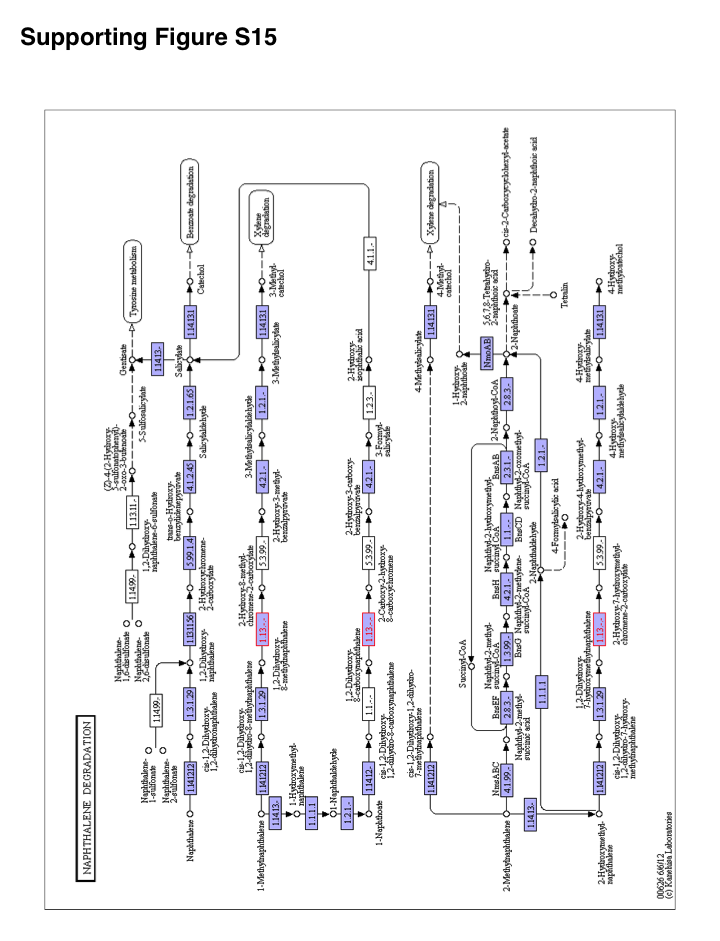

Supplement: Figure S15 — Naftalene degradation. (TIFF) [file pone.0090487.s015.tiff]

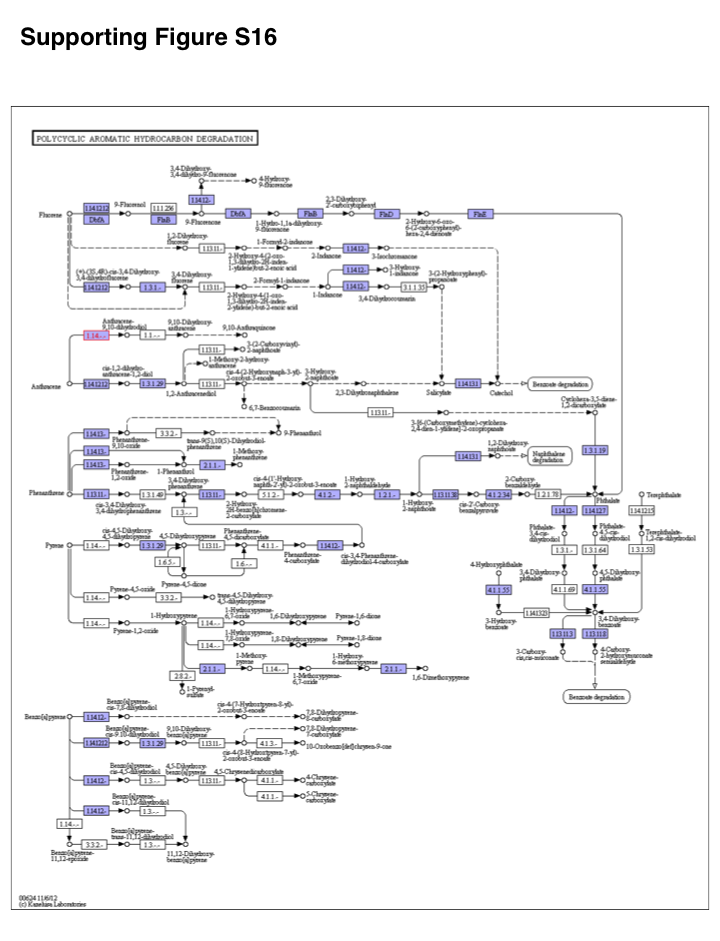

Supplement: Figure S16 — Polycyclic aromatic hydrocarbon degradation. (TIFF) [file pone.0090487.s016.tiff]
