# Supplementary material for: Shedding Some Light over the Floral Metabolism by Arum Lily (Zantedeschia aethiopica) Spathe De Novo Transcriptome Assembly
Source: PLoS One. 2014 Mar 10;9(3):e90487. doi: 10.1371/journal.pone.0090487 (PMC3948674; doi:10.1371/journal.pone.0090487)
Supplement: Table S1 — Cysteine-rich antimicrobial peptides pattern. (PDF) [file pone.0090487.s018.pdf]

Table S1: Cysteine-rich antimicrobial peptides patterns.

Pattern	AMP
C.{6,15}C.{9,31}CC.{8,21}C.C.{13,35}C.{5,18}C	LTP/2S ALBUMIN/ECA 1
C.{5,13}C.{14,20}CC.{8,10}C.{10,32}C	LTP/2S ALBUMIN/ECA 1
C.{4,25}C.{2,12}C.{3,4}C.{3,17}C.{4,32}C.C.{1,6}C	DEFENSIN
C.{2,14}C.{3,5}C.{3,16}C.{4,28}C.C	DEFENSIN
C.{1,8}C.{4,5}CC.{5}C.{6}C.{3,5}C.{3,4}C	HEVEIN
C.{3}C.{3,4}C.{4,32}C.{2,3}C.{3,4}C	THIONIN
CC.{10,11}C.{8,10}C.{5}C.{7,10}C	THIONIN
C.{3}C.{3}C.{7,11}C.{3}C.{2}CC.{2}C.{11}C.{1,2}C	GASA/GAST/SNAKIN
