# Supplementary material for: Shedding Some Light over the Floral Metabolism by Arum Lily (Zantedeschia aethiopica) Spathe De Novo Transcriptome Assembly
Source: PLoS One. 2014 Mar 10;9(3):e90487. doi: 10.1371/journal.pone.0090487 (PMC3948674; doi:10.1371/journal.pone.0090487)
Supplement: Table S2 — Summary of validation parameters for structural three-dimensional models. (PDF) [file pone.0090487.s019.pdf]

Table S2: Summary of validation parameters of structural three-dimensional models

Peptide	Class	Template	Dope Score	Ramachandran Plot		G-Factor	Z-Score (PROSA II)
				Most Favored Regions (%)	Allowed Regions (%)		
3584	LTP	2LJO	-7205,93848	70,4	27,2	-0,10	-4,26
5976	LTP	2LJO	-9223,97461	85,6	8,9	-0,03	-4,35
6924	LTP	1FK5	-9204,11816	85,2	12,3	0,06	-4,34
