# Supplementary material for: Shedding Some Light over the Floral Metabolism by Arum Lily (Zantedeschia aethiopica) Spathe De Novo Transcriptome Assembly
Source: PLoS One. 2014 Mar 10;9(3):e90487. doi: 10.1371/journal.pone.0090487 (PMC3948674; doi:10.1371/journal.pone.0090487)
Supplement: Table S3 — TransDecoder remapping analysis. (PDF) [file pone.0090487.s020.pdf]

Table S3: TransDecoder remapping analysis. Table shows each KO RPKM sum.

KO	Sum (RPKM)
K00001	1111.94894269109
K00002	3595.03714990616
K00003	17.2938995361328
K00008	85.9224967956543
K00010	21.0415999889374
K00011	323.246002197266
K00012	1486.25495529175
K00013	69.557502746582
K00015	42.7831993103027
K00016	9.2248772084713
K00018	93.3740825653076
K00020	306.218171596527
K00021	53.4349012374878
K00024	1734.0251531601
K00025	1734.0251531601
K00026	257.741457223892
K00028	460.476608276367
K00029	191.855697631836
K00030	625.529983520508
K00031	1054.24481472373
K00033	546.764999389648
K00036	2562.43783855438
K00042	195.160900592804
K00052	74.39990234375
K00053	254.628005981445
K00057	117.636476993561
K00058	626.767780303955
K00059	195.024396419525
K00064	45.5119018554688
K00067	9.94359970092773
K00071	2.80611991882324
K00074	263.2080078125
K00075	33.8796005249023
K00079	5.48699009418488
K00081	4.09811007976532
K00083	2582.86506509781
K00084	4.09811007976532
K00085	262.686004638672
K00088	31.5824809074402
K00091	108.604900360107
K00099	213.427993774414
K00100	971.084104597569
K00102	145.142903327942
K00106	83.3154983520508
K00108	26.2812995910645
K00111	159.173004150391

K00117	13.3325996398926
K00118	71.3867988586426
K00119	26.2812995910645
K00120	188.012498855591
K00121	1383.80317491293
K00122	1314.34428632259
K00128	1242.73263835907
K00130	202.036499023438
K00131	121.620601654053
K00133	962.571014404297
K00134	987.286781787872
K00135	100.122201919556
K00139	100.122201919556
K00140	247.345993041992
K00142	6.31156015396118
K00145	32.5996007919312
K00146	324.260987281799
K00161	252.739906311035
K00162	543.752307891846
K00164	93.3287963867188
K00166	20.2229995727539
K00167	145.328994750977
K00207	175.039993286133
K00208	50.8602981567383
K00213	327.784004211426
K00215	40.7970008850098
K00218	27.1517601013184
K00222	45.1472015380859
K00224	289.566785812378
K00225	26.5284004211426
K00227	8.73085021972656
K00228	75.9259033203125
K00231	219.858296394348
K00232	1569.3230805397
K00234	897.281982421875
K00235	349.122985839844
K00249	81.1615982055664
K00252	441.866320252419
K00253	1166.55669403076
K00254	10.0689001083374
K00261	286.351761817932
K00262	730.524677276611
K00264	425.343530654907
K00273	16.1068896055222
K00276	433.397856712341
K00278	29.5466003417969
K00279	22.6740619242191
K00281	193.1090965271
K00284	363.909091949463
K00286	28.7728004455566

K00287	56.4034996032715
K00288	111.559997558594
K00292	43.9546012878418
K00294	74.0639038085938
K00295	290.963012695312
K00297	921.543029785156
K00303	34.6403007507324
K00306	33.6150016784668
K00318	16.6275005340576
K00326	2188.29287719727
K00327	323.603105545044
K00328	134.835103988647
K00329	51.7234001159668
K00331	679.721984863281
K00335	80.8668975830078
K00336	245.251403808594
K00344	634.254364967346
K00355	48.1399002075195
K00356	51.7234001159668
K00360	3.28641998767853
K00365	104.195999145508
K00366	25.7693004608154
K00382	359.314401626587
K00383	243.681297302246
K00384	99.4999980926514
K00392	130.566902160645
K00411	1335.97497558594
K00413	440.134002685547
K00417	351.593002319336
K00422	16.5418707132339
K00423	246.858927249908
K00430	957.781803369522
K00432	1249.00202941895
K00433	1180.77767419815
K00434	3112.31763148308
K00435	229.585998535156
K00441	22.826000213623
K00451	76.3473968505859
K00454	494.509068965912
K00457	7.48529005050659
K00469	297.329299926758
K00470	1.30224001407623
K00472	445.048898696899
K00475	30.0273990631104
K00478	45.8560981750488
K00479	3.30985009670258
K00480	319.315057516098
K00485	182.346229076385
K00487	141.804992675781
K00492	65.7195892333984

K00510	150.608881950378
K00511	209.445999145508
K00514	512.205549240112
K00517	6378.24239832163
K00520	448.45050239563
K00522	1971.94015824795
K00523	43.6950988769531
K00528	26.1156367659569
K00532	40.6459007263184
K00535	230.837005615234
K00540	483.797059595585
K00547	20.7787990570068
K00549	414.565994262695
K00550	25.7133007049561
K00551	67.00830078125
K00555	72.4246753454208
K00556	35.1629981994629
K00558	54.8660273551941
K00559	70.4466018676758
K00565	117.240330457687
K00566	23.1784992218018
K00571	20.8651008605957
K00573	28.2824697494507
K00574	472.929023563862
K00587	44.703498840332
K00588	431.759429454803
K00591	16.2169990539551
K00592	262.625093460083
K00599	413.776711463928
K00600	543.542097091675
K00601	21.1051006317139
K00602	24.6147994995117
K00603	32.94460105896
K00604	12.6052103042603
K00605	153.522003173828
K00606	22.4260005950928
K00609	149.347499847412
K00615	435.0974868536
K00616	592.798004150391
K00620	123.800801992416
K00621	16.1830005645752
K00626	686.458866596222
K00627	442.247901916504
K00630	104.514995574951
K00632	1932.07416629791
K00640	192.232046127319
K00645	137.674297332764
K00648	122.540000915527
K00652	6.3544699549675
K00654	958.86309337616

K00655	31.284999370575
K00658	368.159507751465
K00660	384.556054115295
K00666	220.020998001099
K00670	351.053199768066
K00671	27.4599990844727
K00676	54.1615982055664
K00677	8.85692805051804
K00679	92.7704422473907
K00680	266.591768264771
K00681	94.8388977050781
K00683	392.696990966797
K00685	53.8087997436523
K00688	524.264596939087
K00695	96.0685117840767
K00696	421.687122821808
K00697	691.046360135078
K00700	74.032518863678
K00703	58.0228707790375
K00705	146.167191505432
K00720	2.50026988983154
K00721	68.2841033935547
K00726	59.5755996704102
K00728	163.973007202148
K00729	43.956298828125
K00734	146.849601745605
K00736	49.0663986206055
K00737	174.095199584961
K00748	12.2753251791
K00750	218.945400238037
K00753	77.4065990447998
K00754	21.7915000915527
K00759	144.16708946228
K00760	21.9827995300293
K00761	50.6171989440918
K00763	115.622699737549
K00764	5.8215799331665
K00765	77.6259002685547
K00766	20.5494995117188
K00767	91.0290985107422
K00770	30.4192897081375
K00771	29.8240604400635
K00773	14.4055791497231
K00777	93.5027723312378
K00780	14.442750453949
K00783	21.9722995758057
K00784	41.0851001739502
K00787	237.197992324829
K00789	353.295799255371
K00790	2.01167988777161

K00791	30.2792701721191
K00793	15.444899559021
K00794	174.800003051758
K00797	314.392902374268
K00799	3004.84556293488
K00800	67.7547988891602
K00801	570.731002807617
K00809	64.8417019844055
K00812	52.969898223877
K00813	357.570007324219
K00814	617.610984802246
K00815	174.394972324371
K00817	68.2798004150391
K00818	62.5903015136719
K00819	77.497200012207
K00820	57.2785987854004
K00826	400.403336405754
K00827	407.207234382629
K00830	137.869995117188
K00831	10.7159004211426
K00833	7.43441987037659
K00837	214.847791671753
K00844	538.98366060853
K00847	129.021232128143
K00849	221.3287935853
K00850	253.494522094727
K00851	23.6402523517609
K00852	21.4642095565796
K00854	131.891401052475
K00855	10.5171003341675
K00856	648.755004882812
K00858	157.047596931458
K00859	68.3718032836914
K00860	74.0191292762756
K00861	1867.55606508255
K00863	87.9117669388652
K00864	150.269104003906
K00865	88.9694976806641
K00868	63.2603988647461
K00870	107.772811889648
K00872	259.015991210938
K00873	2478.74663448334
K00875	76.6073503494263
K00876	177.989568591118
K00878	23.468599319458
K00884	26.7917995452881
K00888	195.396101951599
K00889	319.617972612381
K00894	104.539480686188
K00895	657.174892306328

K00898	80.1334991455078
K00899	45.1223983764648
K00901	419.887035608292
K00902	22.7227001190186
K00908	1195.58630537987
K00912	1.73342001438141
K00913	183.499798774719
K00914	31.9681503772736
K00919	148.723299026489
K00921	428.052958488464
K00924	12924.1482329071
K00927	86.0763127207756
K00928	197.434701919556
K00930	35.7896995544434
K00936	17.3449704647064
K00938	27.7408602237701
K00939	442.738444507122
K00940	592.402743339539
K00942	244.52001285553
K00943	89.1917972564697
K00948	294.233309745789
K00949	103.499911546707
K00951	673.091468811035
K00953	94.0886993408203
K00960	6.47763013839722
K00962	62.0818737149239
K00963	164.945007324219
K00965	23.7576007843018
K00966	390.194007873535
K00967	69.6691989898682
K00968	182.819497108459
K00970	28.31656062603
K00971	348.633905410767
K00972	32.3348999023438
K00974	14.7978000640869
K00975	445.289862751961
K00979	63.5396995544434
K00981	139.121021270752
K00985	62.847900390625
K00986	1.8826299905777
K00987	15.298999786377
K00991	105.68399810791
K00993	278.554992675781
K00995	67.6854019165039
K00999	56.1452004909515
K01001	217.852909088135
K01003	9.69289970397949
K01006	7.72996997833252
K01007	86.0560989379883
K01011	173.078895568848

K01012	47.1058006286621
K01046	289.712005615234
K01047	21.6636803150177
K01051	627.193214803934
K01052	42.5297012329102
K01054	436.43665933609
K01056	58.6810593605042
K01057	167.518506526947
K01058	65.328501701355
K01061	345.299970030785
K01066	288.241594314575
K01068	70.331298828125
K01069	257.652832388878
K01070	149.136001586914
K01072	13.1330995559692
K01074	240.32600402832
K01076	70.331298828125
K01078	273.729403734207
K01079	230.141998291016
K01080	45.9417881965637
K01081	73.9580616950989
K01082	35.5680289268494
K01087	706.455159187317
K01090	2555.01885318756
K01091	41.9607009887695
K01092	72.056999206543
K01099	145.446399927139
K01100	7.78542017936707
K01101	534.943651556969
K01102	564.30236530304
K01103	152.239896774292
K01104	402.935398101807
K01106	6.50419998168945
K01110	226.925825595856
K01113	84.8121067285538
K01114	162.343075871468
K01115	813.059012115002
K01126	77.6097893714905
K01142	1.44562995433807
K01143	124.880996704102
K01144	67.0341987609863
K01147	23.5802993774414
K01148	47.0878396034241
K01157	47.140998840332
K01164	19.9186898469925
K01166	254.305999755859
K01170	84.0037002563477
K01174	122.290302276611
K01175	317.032125473022
K01176	844.956832408905

K01177	1177.93504929543
K01179	440.991135299206
K01180	6.29163980484009
K01181	23.4122009277344
K01183	20.2042492032051
K01184	22.9551563858986
K01187	147.742810368538
K01188	589.663776315749
K01190	189.197985649109
K01191	78.3560590744019
K01192	22.8105792999268
K01193	1177.00646275282
K01194	94.8274974822998
K01196	4.29328012466431
K01200	18.8871002197266
K01205	69.3508996963501
K01206	11.647500038147
K01209	448.728603363037
K01210	11.57493019104
K01213	76.6707504987717
K01227	58.0758018493652
K01228	21.221700668335
K01230	405.225083351135
K01231	96.2213010787964
K01238	119.987998962402
K01243	16.8676500320435
K01244	139.296005249023
K01246	715.408468425274
K01247	10.4867599010468
K01249	331.135902404785
K01251	649.639007568359
K01253	77.0313410758972
K01254	55.1979703903198
K01255	339.769012451172
K01256	282.744901657104
K01259	62.3271994590759
K01261	115.610000610352
K01262	231.561677455902
K01265	668.618602752686
K01267	134.527896881104
K01276	46.1181983947754
K01277	14.938099861145
K01278	37.7005996704102
K01280	27.0227700471878
K01285	262.899390220642
K01301	8.86667990684509
K01303	27.0028991699219
K01304	111.718002319336
K01307	42.7018013000488
K01322	18.1359996795654

K01330	0.961641013622284
K01338	503.633911132812
K01354	93.5760941505432
K01358	2104.98820495605
K01362	828.261652648449
K01363	1008.96997070312
K01365	9.78134614229202
K01366	1445.90074443817
K01367	20.2276000976562
K01369	97.5084959864616
K01373	855.630104064941
K01376	2013.46166592836
K01381	46.1365995407104
K01392	5.23423004150391
K01404	54.8471093177795
K01408	208.084874153137
K01409	40.7531900405884
K01410	23.1444796323776
K01411	44.6912994384766
K01412	740.777871608734
K01414	356.166481018066
K01415	72.6543006896973
K01417	70.3814010620117
K01423	96.7320995330811
K01425	94.8869018554688
K01426	78.5847117900848
K01427	3.82495999336243
K01431	148.994003295898
K01433	39.7485585212708
K01436	24.5156002044678
K01438	79.4928970336914
K01444	28.5352001190186
K01454	30.5432638525963
K01455	13.8861799240112
K01456	125.697298049927
K01462	86.6686117649078
K01463	50.2170633673668
K01464	186.211692810059
K01465	44.1408996582031
K01466	45.3946990966797
K01469	19.9506196975708
K01476	197.574806213379
K01480	64.4304962158203
K01487	336.501800537109
K01488	93.0955991744995
K01489	41.5629005432129
K01490	14.2059001922607
K01491	129.532098770142
K01493	1.96501004695892
K01495	81.8081970214844

K01500	4.07294988632202
K01501	37.5693998336792
K01505	38.1837301254272
K01506	38.8586006164551
K01507	1663.59610939026
K01509	241.717008113861
K01510	384.195499420166
K01511	3.3497200012207
K01512	12.6823997497559
K01513	44.6079912185669
K01514	62.3777121007442
K01517	12.6702995300293
K01520	1.43854999542236
K01522	89.4982986450195
K01527	382.466209411621
K01528	496.755805969238
K01529	394.068988293409
K01530	347.714001536369
K01531	3.24745988845825
K01533	173.203908443451
K01534	75.8805999755859
K01535	334.567261278629
K01537	470.410005331039
K01551	166.212997436523
K01552	15.2299003601074
K01553	37.6152670383453
K01554	25.9489898681641
K01555	155.590698242188
K01557	118.872001647949
K01563	8.52956962585449
K01567	302.015951156616
K01568	7548.25388336182
K01580	596.985506057739
K01582	26.1177005767822
K01583	106.028299331665
K01586	42.0555992126465
K01587	0.281094998121262
K01590	88.9422912597656
K01592	48.1094210147858
K01593	48.1094210147858
K01595	614.375405311584
K01597	155.885097503662
K01598	76.6517405509949
K01599	720.22367477417
K01601	55.6173000335693
K01602	195.360992431641
K01609	35.3069000244141
K01610	37.6196913719177
K01611	1044.66732633114
K01613	101.342103004456

K01618	80.3270034790039
K01620	164.621600151062
K01623	1344.61526447535
K01625	2.77722001075745
K01626	88.4545974731445
K01627	24.5664005279541
K01633	56.3951988220215
K01634	261.424705505371
K01640	70.3460998535156
K01641	825.66202878952
K01647	603.505996704102
K01648	608.043296813965
K01649	29.0123130679131
K01652	28.5160999298096
K01653	238.789505004883
K01657	14.3488998413086
K01658	129.552993774414
K01661	4.78654003143311
K01662	69.4405794143677
K01663	32.5086002349854
K01669	227.496101617813
K01674	16.8415200710297
K01679	49.1357002258301
K01681	217.294994354248
K01687	17.193000793457
K01689	1774.44003629684
K01692	329.203701019287
K01695	207.697601318359
K01696	54.322699546814
K01698	202.039001464844
K01703	159.268005371094
K01704	140.667007446289
K01710	326.130996704102
K01711	102.319000244141
K01714	62.4940986633301
K01718	55.1633005142212
K01719	38.7613997459412
K01723	100.877501487732
K01725	73.7807998657227
K01728	20.3990993499756
K01733	2454.1298828125
K01735	143.404998779297
K01736	138.304992675781
K01738	818.377481460571
K01739	316.317993164062
K01741	14.6663298606873
K01743	29.9097003936768
K01746	32.94460105896
K01749	201.557998657227
K01750	3.72233009338379

K01754	5032.86074447632
K01755	43.9669990539551
K01756	42.9420013427734
K01757	204.199421644211
K01758	8.92618012428284
K01759	581.25860440731
K01760	51.2693500518799
K01761	184.475997924805
K01768	164.518801689148
K01770	247.236999511719
K01772	619.140014648438
K01778	110.579002380371
K01779	59.0435981750488
K01783	451.625007629395
K01784	794.608372449875
K01785	457.966105461121
K01792	419.128705978394
K01798	5.19784021377563
K01800	151.412994384766
K01802	2456.71159756184
K01803	1535.47601318359
K01805	593.879028320312
K01807	274.135662913322
K01809	198.876007080078
K01810	333.186547994614
K01814	10.5544004440308
K01817	46.3526000976562
K01823	144.425003051758
K01824	189.457992553711
K01829	392.087089538574
K01834	1423.5791965723
K01835	425.268894195557
K01836	129.803839683533
K01837	1.20511996746063
K01838	274.779459476471
K01840	651.803607940674
K01845	178.961086392403
K01850	198.285999298096
K01852	37.0511016845703
K01853	37.0511016845703
K01855	25.0858072042465
K01858	113.721000671387
K01859	95.0971984863281
K01866	100.317203521729
K01867	123.371720314026
K01868	452.701705932617
K01869	93.7886674404144
K01870	171.084202408791
K01872	100.957391738892
K01873	59.2445497512817

K01874	366.405298709869
K01875	166.500200271606
K01876	217.869386196136
K01880	148.459800720215
K01881	372.774936437607
K01883	251.890099525452
K01885	111.554298400879
K01886	53.9564018249512
K01887	184.582595825195
K01889	124.651500701904
K01890	44.0453987121582
K01892	82.1538009643555
K01893	230.499002456665
K01895	212.571998596191
K01897	1633.92917633057
K01899	547.232986450195
K01900	458.702987670898
K01904	31.3710913956165
K01913	213.942699432373
K01915	748.143787384033
K01918	112.929100036621
K01919	230.647694885731
K01920	145.567510128021
K01921	3.78663003444672
K01922	55.1226997375488
K01923	21.864200592041
K01925	19.1807101964951
K01928	12.3961100578308
K01930	158.455497205257
K01931	326.607275485992
K01934	126.207908153534
K01937	296.17297077179
K01938	111.559997558594
K01939	78.6592025756836
K01940	156.610500335693
K01942	22.6646995544434
K01945	9.10822010040283
K01950	123.870899200439
K01951	64.8839015960693
K01952	36.5932404994965
K01953	2101.79632246494
K01955	41.5807499885559
K01956	82.0192031860352
K01957	2.39006996154785
K01961	134.588800430298
K01962	221.666105270386
K01963	1.99987995624542
K01968	266.567202568054
K01969	268.358800888062
K01976	134.192380070686

K02006	23.6324005126953
K02008	40.2234992980957
K02021	27.7636301517487
K02055	15.8754997253418
K02057	39.7361488342285
K02065	75.3479387760162
K02066	77.3753967285156
K02067	33.9031982421875
K02068	9.40038967132568
K02069	138.539001464844
K02078	7.38790988922119
K02083	265.280006408691
K02087	61.837100982666
K02090	61.837100982666
K02109	12.7496995925903
K02111	28.5755591392517
K02112	5.83893990516663
K02113	260.091003417969
K02114	3.70797991752625
K02115	22.8798999786377
K02116	125.741996765137
K02118	837.611999511719
K02132	23.0259990692139
K02133	430.401046991348
K02134	351.961990356445
K02136	564.462701797485
K02137	718.637023925781
K02138	585.257995605469
K02140	1279.70575714111
K02144	242.158004760742
K02145	652.830792874098
K02146	260.25
K02147	1010.62800598145
K02148	262.881988525391
K02149	156.343994140625
K02150	621.886996984482
K02152	1240.93005371094
K02154	444.750392913818
K02155	913.116027832031
K02160	93.8839988708496
K02180	18.3260998725891
K02183	2931.14917159081
K02193	14.4545001983643
K02201	31.3413200378418
K02202	52.8250007629395
K02206	61.837100982666
K02208	187.38809967041
K02210	0.921788990497589
K02212	0.71779602766037
K02218	1005.55814671516

K02221	31.3178901672363
K02234	43.8869787454605
K02257	147.334302902222
K02258	27.0088996887207
K02259	35.9826011657715
K02263	270.068359851837
K02265	501.855987548828
K02266	1052.4999294281
K02267	128.268997192383
K02291	96.3131999969482
K02292	12.4692001342773
K02293	73.2534027099609
K02294	2.63740992546082
K02295	53.0517501831055
K02302	19.5830001831055
K02303	19.5830001831055
K02320	3.99423003196716
K02321	10.8554501533508
K02324	22.6996411681175
K02327	8.1304098367691
K02328	3.5166699886322
K02331	7.41769981384277
K02335	53.9692702293396
K02342	71.6233601570129
K02343	7.91045999526978
K02349	6.66744804382324
K02350	60.696830034256
K02355	377.066898345947
K02356	54.4181808233261
K02357	41.8380012512207
K02358	78.0438995361328
K02365	7.23823302984238
K02366	9.65869045257568
K02367	16.8613495826721
K02370	11.7904996871948
K02371	17.660400390625
K02372	220.388006210327
K02377	81.2914962768555
K02427	4.15826988220215
K02433	300.395785927773
K02434	54.1084003448486
K02437	124.366877555847
K02438	255.191000938416
K02469	24.0260291099548
K02470	21.4946799278259
K02471	20.9941005706787
K02485	60.0263996124268
K02492	14.2671003341675
K02493	5.50079011917114
K02495	1.88182896375656

K02503	466.941612243652
K02510	9.68663024902344
K02516	33.2883005142212
K02519	54.9852981567383
K02520	106.660003662109
K02527	12.0261201858521
K02528	13.149299621582
K02535	4.56780004501343
K02536	33.2911987304688
K02540	0.74881899356842
K02541	2.41955995559692
K02548	5.33853006362915
K02549	55.8893013000488
K02552	3.15228009223938
K02553	550.259498596191
K02563	7.78847980499268
K02575	0.9966099858284
K02604	13.532600402832
K02606	2.14514994621277
K02634	5.09998989105225
K02636	33.622200012207
K02638	13.0290002822876
K02639	3392.7094669342
K02641	302.360103607178
K02687	6.45888996124268
K02689	16.0636859238148
K02690	5.38440990447998
K02694	107.352996826172
K02698	51.6934013366699
K02701	30.8474998474121
K02704	4.27963018417358
K02716	590.017028808594
K02717	496.394008636475
K02721	388.365386962891
K02723	134.351265907288
K02725	260.160003662109
K02726	271.123992919922
K02727	203.921005249023
K02728	122.391998291016
K02729	569.746002197266
K02730	346.915985107422
K02731	178.248701095581
K02732	222.908004760742
K02734	255.175994873047
K02735	203.554000854492
K02736	189.563995361328
K02737	353.498001098633
K02738	269.737293243408
K02739	482.409889221191
K02830	12.7897996902466

K02834	91.8583984375
K02835	27.7892999649048
K02836	130.037307977676
K02838	159.068803787231
K02861	45.8620986938477
K02863	367.427324771881
K02864	49.0643997192383
K02865	971.453253626823
K02866	869.650119066238
K02867	38.1350994110107
K02868	276.689211606979
K02870	216.61934697628
K02871	370.514507293701
K02872	2.09846997261047
K02873	345.456114292145
K02874	22.4305000305176
K02875	467.686660885811
K02876	187.700798034668
K02877	362.46809899807
K02879	193.824895858765
K02880	931.0242228508
K02881	165.058272838593
K02882	0.69718998670578
K02883	679.730275690556
K02884	307.285003662109
K02885	728.101632118225
K02886	64.8311996459961
K02887	19.7987995147705
K02888	189.033004760742
K02889	504.390065670013
K02891	266.675994873047
K02893	798.085699319839
K02894	1281.81714451313
K02895	373.137096405029
K02896	1040.36229717731
K02897	71.3493013381958
K02898	823.176203370094
K02899	182.697006225586
K02900	2.21650004386902
K02901	317.560492217541
K02903	732.745025634766
K02904	404.002990722656
K02906	162.008102416992
K02909	82.3713989257812
K02910	608.869300723076
K02912	968.044982910156
K02915	1981.82995605469
K02917	1587.02655887604
K02918	406.929722011089
K02920	721.017018318176

K02925	1212.45542800426
K02926	295.778991699219
K02929	799.118988037109
K02930	235.118055105209
K02931	388.542999267578
K02932	837.608299612999
K02933	537.147994995117
K02934	699.019097328186
K02935	153.297899246216
K02936	827.90755546093
K02937	577.268424272537
K02938	420.857928752899
K02939	174.373003005981
K02940	1242.47500634193
K02941	800.739530205727
K02942	605.321014404297
K02943	1025.23772192001
K02945	394.200292110443
K02946	195.985992431641
K02949	440.301318049431
K02951	580.518945097923
K02953	334.717010498047
K02954	57.6285018920898
K02955	590.128070116043
K02957	789.173281669617
K02958	867.442993164062
K02960	513.992605209351
K02961	30.2583999633789
K02964	649.207501411438
K02965	88.9452972412109
K02966	927.389705777168
K02969	359.217738986015
K02971	505.502014160156
K02973	1054.2967416048
K02974	405.235219955444
K02975	588.27430999279
K02977	2.22612804174423
K02979	6.30544996261597
K02981	217.708999633789
K02983	0.286606997251511
K02984	533.787144899368
K02985	335.51047873497
K02986	1.35459005832672
K02987	419.439419865608
K02988	196.744995117188
K02989	256.166278958321
K02990	138.962997436523
K02991	792.924178123474
K02992	255.126998901367
K02993	1.01627004146576

K02995	323.084361195564
K02996	533.163005828857
K02997	882.281112253666
K02998	421.573881983757
K02999	27.2739605903625
K03000	32.7112998962402
K03002	10.3640003204346
K03005	28.3258991241455
K03006	46.9356755316257
K03008	150.6756067276
K03010	131.239601373672
K03011	88.8003997802734
K03013	158.211101531982
K03014	459.199005126953
K03015	576.076591491699
K03016	267.985533952713
K03017	88.2735977172852
K03018	10.7470703125
K03020	49.1101989746094
K03021	11.8010997772217
K03022	25.5412006378174
K03023	46.3787002563477
K03024	6.78423976898193
K03026	32.7501804828644
K03027	14.2119998931885
K03028	108.947769165039
K03029	296.410705566406
K03030	230.251998901367
K03031	335.794006347656
K03032	187.937994003296
K03033	282.276596069336
K03035	214.111999511719
K03036	120.578002929688
K03037	164.281005859375
K03038	221.063003540039
K03039	357.740997314453
K03040	5.54146003723145
K03043	1.84174001216888
K03046	4.84173011779785
K03061	1581.6880607605
K03062	455.934997558594
K03063	208.300003051758
K03064	117.560997009277
K03065	182.727005004883
K03066	49.5999984741211
K03070	53.4547591209412
K03076	12.7839002609253
K03083	602.13960647583
K03086	2.44468998908997
K03087	21.4604704380035

K03093	8.75973987579346
K03094	1244.70416116714
K03097	351.226997375488
K03098	35.0144996643066
K03100	358.820789337158
K03104	110.850997924805
K03105	427.734008789062
K03106	314.449199676514
K03107	64.9386978149414
K03108	87.2253017425537
K03109	54.9217987060547
K03110	65.1527004241943
K03111	16.9587903022766
K03113	2818.59301757812
K03115	256.276911735535
K03116	258.446990966797
K03118	179.473999023438
K03120	69.1643981933594
K03122	116.046997070312
K03123	178.349201202393
K03124	172.688670158386
K03125	30.615870475769
K03126	66.0779099464417
K03127	30.1026202440262
K03128	49.2798302173615
K03130	29.6202993392944
K03131	70.5040969848633
K03132	71.0442962646484
K03135	31.9745998382568
K03136	78.0832014083862
K03137	239.821055412292
K03138	238.981094360352
K03139	93.8600006103516
K03140	240.564601898193
K03141	30.4943099021912
K03142	25.8215999603271
K03143	30.3302011489868
K03144	52.614138841629
K03145	55.1316986083984
K03147	110.361999511719
K03152	120.209247112274
K03155	249.622072696686
K03163	88.1620998382568
K03165	360.145946800709
K03167	37.7045001983643
K03168	8.10414981842041
K03169	153.074398040771
K03177	48.7758712768555
K03178	204.263998985291
K03183	58.1079205274582

K03188	288.343561410904
K03189	97.4539031982422
K03190	60.5163993835449
K03217	1055.63645386696
K03231	4098.58376014233
K03232	243.214174151421
K03233	516.286047816277
K03234	594.925176203251
K03236	516.848999023438
K03237	100.147003173828
K03238	346.317993164062
K03239	49.0422999858856
K03240	114.510299682617
K03241	75.1773986816406
K03242	195.165596008301
K03243	177.329498291016
K03245	42.035099029541
K03246	285.299011230469
K03247	437.286987304688
K03248	750.797819137573
K03249	61.5617980957031
K03250	694.56201171875
K03251	28.2915000915527
K03252	56.9730987548828
K03253	207.197410702705
K03254	170.435400009155
K03255	20.7150001525879
K03256	6.35669994354248
K03257	1181.93948459625
K03259	1557.17600250244
K03260	606.16202673316
K03262	832.630989074707
K03263	534.414254456758
K03264	154.570297241211
K03265	149.789993286133
K03267	110.350997924805
K03281	54.7837114334106
K03283	4497.10841315985
K03292	57.910099029541
K03294	175.503379821777
K03301	432.016090393066
K03304	14.0845701694489
K03319	89.4394989013672
K03320	36.5256996154785
K03321	57.4641990661621
K03322	21.4943714141846
K03327	618.846621632576
K03347	206.579620718956
K03348	6.33640992641449
K03349	6.37740993499756

K03350	11.2560195922852
K03351	3.18653011322021
K03352	4.90098989009857
K03353	2.98379993438721
K03354	18.7530002593994
K03355	6.36994981765747
K03357	40.6343994140625
K03361	44.0503997802734
K03362	2.17650008201599
K03363	152.895749092102
K03364	515.602832794189
K03384	4.33634704351425
K03386	1535.00182831287
K03403	4.43614000082016
K03404	42.0805015563965
K03405	60.0873985290527
K03417	2295.97998046875
K03424	46.566499710083
K03426	45.8432998657227
K03428	2.32652997970581
K03434	30.397539973259
K03437	5.01599979400635
K03438	27.0608997344971
K03439	103.080927371979
K03453	228.80324369669
K03454	1151.91044461727
K03455	199.00071811676
K03456	269.096008300781
K03457	103.006050109863
K03469	28.6372900009155
K03495	36.3086878061295
K03500	4.51259994506836
K03501	20.7352695465088
K03504	37.5335006713867
K03509	6.27459001541138
K03511	42.7901992797852
K03512	1.74214005470276
K03514	75.4683594703674
K03515	112.237003564835
K03517	67.4324016571045
K03521	57.6156005859375
K03522	38.2061996459961
K03526	358.390014648438
K03527	553.645004272461
K03531	136.647199630737
K03537	15.6007995605469
K03538	34.6342010498047
K03539	29.3688402175903
K03541	709.6669921875
K03542	46.3305015563965

K03544	308.66496348381
K03545	34.0265998840332
K03549	2245.75520306826
K03553	57.5511906147003
K03564	12.6680002212524
K03574	59.0839083194733
K03575	3.44297003746033
K03593	120.287979841232
K03595	152.35279917717
K03596	151.222501754761
K03609	17.0951995849609
K03626	120.256625235081
K03627	2294.46538352966
K03635	3.35111999511719
K03639	53.6867738962173
K03644	92.3027992248535
K03648	11.9188003540039
K03650	11.6035599708557
K03652	11.5403499603271
K03654	41.6196002960205
K03655	4.5734601020813
K03661	207.572998046875
K03665	38.5558013916016
K03671	5350.33284473419
K03676	661.170997619629
K03678	13.3662004470825
K03679	26.4440002441406
K03680	107.644397735596
K03681	95.2737584114075
K03686	716.624580144882
K03687	536.696300506592
K03688	344.509502410889
K03691	76.183198928833
K03695	186.381324231625
K03696	813.036987304688
K03699	146.815998077393
K03707	75.1799011230469
K03714	17.4752006530762
K03715	58.8456099033356
K03723	19.6478395462036
K03731	7.34096002578735
K03750	74.7049007415771
K03754	39.2024002075195
K03767	738.122284531593
K03768	1275.70249998569
K03772	12.1408996582031
K03781	1436.41900634766
K03787	87.3318023681641
K03797	79.9765303134918
K03798	1151.27291712165

K03800	20.1219997406006
K03801	54.9339008331299
K03809	644.560205459595
K03834	3.08477008342743
K03841	271.555104374886
K03842	107.114501953125
K03843	43.9597015380859
K03844	32.617849946022
K03845	28.9380596876144
K03846	21.2371997833252
K03847	15.7341003417969
K03848	16.7699704170227
K03849	12.4853000640869
K03850	44.6804401874542
K03853	20.2276000976562
K03857	567.799272119999
K03859	301.521697998047
K03860	20.2581996917725
K03861	24.6837005615234
K03869	85.2924003601074
K03872	267.471008300781
K03875	95.6965026855469
K03876	8.01933002471924
K03879	3.32475996017456
K03884	4.03601980209351
K03885	336.58616065979
K03919	9.46354007720947
K03921	240.254752397537
K03926	64.5343198776245
K03934	245.251403808594
K03936	590.452324867249
K03937	253.231994628906
K03940	679.721984863281
K03941	602.296981811523
K03942	570.396606445312
K03943	477.773010253906
K03949	179.227996826172
K03950	144.507995605469
K03952	230.772003173828
K03953	138.164001464844
K03955	328.214902877808
K03965	258.207000732422
K03966	328.778015136719
K03969	190.429520130157
K03977	75.0303993225098
K03978	11.8038601875305
K03979	229.207459449768
K04035	59.8269996643066
K04040	22.7418003082275
K04043	1366.94924640656

K04077	2572.99923580885
K04078	1560.47660446167
K04079	738.954788386822
K04082	44.828498840332
K04120	9.06954956054688
K04121	33.4344961047173
K04122	19.4969396591187
K04123	1.95489001274109
K04124	71.4765014648438
K04125	4.39607107639313
K04127	91.5038986206055
K04228	9.26159000396729
K04345	500.602880001068
K04354	362.632612794638
K04368	550.570611476898
K04371	701.281601905823
K04374	0.469125002622604
K04382	283.069400787354
K04392	268.092971801758
K04417	24.8531999588013
K04418	207.441802978516
K04422	123.852996826172
K04424	627.83914655447
K04427	94.8869018554688
K04437	6.94747018814087
K04457	84.7126998901367
K04459	102.078895568848
K04460	161.567195892334
K04461	84.7126998901367
K04482	4.16118001937866
K04485	4.22038984298706
K04487	80.2944030761719
K04488	690.7490234375
K04496	30.1585006713867
K04498	16.2747898101807
K04499	16.7609004974365
K04505	13.6372995376587
K04506	326.651203155518
K04507	273.241607666016
K04508	97.8936996459961
K04512	85.6488099098206
K04513	0.323383986949921
K04518	129.070877552032
K04520	1.28575003147125
K04523	221.499898910522
K04524	0.504033029079437
K04532	71.8110990524292
K04536	45.6885986328125
K04551	39.9519996643066
K04554	242.988192081451

K04563	61.837100982666
K04564	423.110092163086
K04565	2659.10865783691
K04567	72.3997497558594
K04569	33.3026008605957
K04602	56.8749008178711
K04640	316.318323135376
K04646	325.517101287842
K04649	75.1209030151367
K04650	25.0564994812012
K04681	47.3396987915039
K04683	60.0931087732315
K04688	108.321998596191
K04705	166.473495483398
K04706	70.6442613601685
K04708	3.51732993125916
K04709	37.9886016845703
K04711	95.6651992797852
K04712	23.4109992980957
K04713	494.835494995117
K04715	54.0287598371506
K04716	60.6525402069092
K04718	105.754429101944
K04728	615.427226066589
K04733	3326.41453355551
K04751	58.5578002929688
K04752	58.5578002929688
K04755	54.2432398796082
K04773	54.5211982727051
K04794	56.3488998413086
K04797	94.4794006347656
K04798	110.450996398926
K04799	54.8292999267578
K04802	4.90742015838623
K04883	110.860000610352
K04909	18.7383003234863
K04910	13.9378004074097
K05016	241.693705022335
K05021	121.706203460693
K05022	76.5511016845703
K05024	76.5511016845703
K05025	76.5511016845703
K05084	72.0019989013672
K05236	534.218231201172
K05275	1295.42137336731
K05277	62.2016070485115
K05278	101.305797576904
K05279	183.329803466797
K05280	185.562562942505
K05283	30.7455005645752

K05284	11.6878597736359
K05285	7.16684007644653
K05286	19.2829598784447
K05287	12.1014995574951
K05288	42.2753009796143
K05289	60.6766014099121
K05290	191.884498596191
K05291	43.65149974823
K05292	48.9475871920586
K05293	24.8278007507324
K05298	84.9586029052734
K05302	6.13169002532959
K05305	25.6235008239746
K05309	26.6126003265381
K05310	17.367870092392
K05349	1546.62557220459
K05350	319.567206859589
K05356	305.691986083984
K05366	8.01951003074646
K05387	266.895588874817
K05389	20.7904102802277
K05391	467.25243294239
K05395	48.1399002075195
K05396	14.8532100915909
K05399	126.535130023956
K05516	260.736466884613
K05520	97.5991020202637
K05535	71.8992004394531
K05539	5.81391000747681
K05542	9.82419967651367
K05543	23.0196399688721
K05544	54.2806015014648
K05545	9.82419967651367
K05546	147.220897674561
K05575	1.29928004741669
K05577	5.4209201335907
K05579	2.97718000411987
K05581	5.4698600769043
K05584	5.87788009643555
K05585	10.8425998687744
K05592	237.276720523834
K05605	117.349697113037
K05607	55.1217002868652
K05609	424.614017009735
K05610	131.979797363281
K05643	11.1111221313477
K05648	7.29349994659424
K05655	4.31959998607635
K05656	15.89035987854
K05657	4.93018007278442

K05658	238.033983469009
K05663	25.5608997344971
K05664	10.5342998504639
K05665	179.937608957291
K05666	315.45115852356
K05667	160.514449357986
K05668	119.160040855408
K05671	49.701099395752
K05673	103.368452072144
K05674	4.43347990512848
K05677	67.6767997741699
K05680	71.2293977737427
K05681	1141.36479246616
K05692	2754.63441419601
K05715	74.6334816217422
K05732	71.4860000610352
K05743	16.2339000701904
K05747	23.6354999542236
K05749	22.7201995849609
K05750	28.4900989532471
K05754	164.550994873047
K05755	10.019700050354
K05756	204.449005126953
K05757	89.4590015411377
K05758	95.3267412185669
K05759	382.790985107422
K05765	1985.79498672485
K05766	187.587295532227
K05768	210.495700061321
K05794	45.9682006835938
K05841	170.836183190346
K05849	27.2702698707581
K05853	228.953510761261
K05857	280.744916796684
K05858	281.432907104492
K05863	1719.01062965393
K05864	27.7390003204346
K05882	6.28918981552124
K05885	102.034253418446
K05894	333.739948272705
K05906	8.72272968292236
K05907	2.8696300983429
K05917	237.979995727539
K05925	66.6275005340576
K05928	69.7251968383789
K05929	17.2982997894287
K05931	86.7132034301758
K05933	60.9865307807922
K05954	58.0644016265869
K05955	101.169902801514

K05956	167.497226715088
K05962	15.9176204204559
K05982	35.0431495904922
K05986	85.856201171875
K05989	85.5203018188477
K05993	110.05374789238
K06001	11.6323003768921
K06013	195.004104614258
K06016	54.380199432373
K06022	20.2133197784424
K06027	39.0741996765137
K06041	33.648998260498
K06062	26.6695003509521
K06063	53.7703018188477
K06066	39.3795013427734
K06067	127.186690270901
K06072	32.7706985473633
K06085	32.6130981445312
K06096	7.02496004104614
K06100	43.0260567963123
K06101	37.2634010314941
K06110	35.2536010742188
K06111	33.4600982666016
K06115	28.4418992996216
K06118	28.0107002258301
K06119	48.1535987854004
K06123	24.3931999206543
K06125	7.13259983062744
K06126	233.776397705078
K06127	56.2518005371094
K06129	14.4146003723145
K06130	204.412105560303
K06133	12.418380022049
K06147	75.835560798645
K06158	338.250991821289
K06167	23.0954999923706
K06168	14.177300453186
K06170	67.3106002807617
K06171	24.2784996032715
K06172	130.231994628906
K06173	69.4871606826782
K06174	269.562004089355
K06179	29.1644291877747
K06180	20.4949402809143
K06184	32.8479995727539
K06185	2119.19364929199
K06189	2.85245990753174
K06195	48.9798011779785
K06196	90.2385025024414
K06207	101.048198699951

K06210	54.5432004928589
K06215	100.346000671387
K06221	0.737551987171173
K06236	77.0241012573242
K06268	447.922902941704
K06269	521.4928150177
K06272	12.3318004608154
K06276	76.3311004638672
K06287	71.3601505756378
K06316	16.592139840126
K06324	37.8494987487793
K06390	26.0656299591064
K06413	7.67081022262573
K06442	18.8980007171631
K06443	78.9232025146484
K06444	27.7300705909729
K06515	29.6404991149902
K06611	477.594352424145
K06620	9.06424999237061
K06628	6.23796987533569
K06630	1459.52415573597
K06634	136.780097961426
K06636	30.6998805999756
K06638	11.6280002593994
K06640	5.78834295272827
K06653	71.8992004394531
K06662	10.7448997497559
K06664	35.3566017150879
K06669	46.2556991577148
K06670	1.90383005142212
K06671	30.4009990692139
K06672	58.9147005081177
K06676	77.1069782376289
K06678	0.491916000843048
K06683	16.2301998138428
K06685	382.928409576416
K06689	6520.27793419361
K06692	28.7586002349854
K06693	27.3285999298096
K06694	86.9897081851959
K06699	55.0897998809814
K06700	65.1652984619141
K06867	554.2239112854
K06872	15.1651000976562
K06874	121.876300811768
K06875	295.308013916016
K06883	844.88729763031
K06885	5.87544012069702
K06889	405.170224189758
K06890	686.5830078125

K06891	112.200996398926
K06892	881.740831553936
K06896	33.2703018188477
K06900	30.8513004779816
K06901	3.1519900560379
K06911	9.68884992599487
K06916	227.663990259171
K06927	5.76557016372681
K06928	15.4725999832153
K06940	119.231002807617
K06941	137.940898895264
K06942	328.388893127441
K06943	102.653302311897
K06944	684.284175872803
K06945	127.476997375488
K06947	24.3183994293213
K06948	19.7175550460815
K06949	49.1208992004395
K06950	9.27132034301758
K06955	163.637802124023
K06961	94.5118026733398
K06962	44.3259010314941
K06963	49.7593994140625
K06965	53.1166000366211
K06966	264.138391017914
K06969	18.1331748366356
K06972	76.3665008544922
K06980	59.4747009277344
K06981	36.2178001403809
K06990	78.7301025390625
K06995	68.3414993286133
K06997	520.315979003906
K06998	51.405101776123
K07007	2.65307796001434
K07011	1.93313002586365
K07015	74.8672103881836
K07018	14.4858999252319
K07019	23.0880002975464
K07020	1.3972100019455
K07021	13.5796003341675
K07023	129.816898345947
K07024	152.366697311401
K07025	252.537070512772
K07029	128.142200469971
K07036	11.4800996780396
K07042	6.25538015365601
K07046	22.6940002441406
K07047	6.04330015182495
K07052	118.441157817841
K07053	37.3768005371094

K07055	133.686499595642
K07056	9.47428035736084
K07059	254.009199142456
K07071	34.1996994018555
K07088	558.057893693447
K07095	452.642105102539
K07107	11.7861995697021
K07114	202.044994354248
K07119	768.023597717285
K07126	72.3374004364014
K07130	176.74299621582
K07137	10.6548004150391
K07140	35.7498006820679
K07146	33.232608795166
K07151	82.8595008850098
K07152	28.1908392906189
K07178	21.4123992919922
K07179	21.6771602630615
K07182	489.792695999146
K07192	48.1663017272949
K07195	361.597654342651
K07198	443.229949444532
K07199	457.660110473633
K07203	22.2513008117676
K07204	57.9026985168457
K07226	87.2109985351562
K07232	104.290539264679
K07238	23.8024997711182
K07243	64.7067031860352
K07249	324.260987281799
K07252	95.4051036834717
K07253	795.56508731842
K07263	65.4086990356445
K07276	28.7192001342773
K07277	194.545223236084
K07297	93.999498128891
K07300	246.908070087433
K07304	136.430503845215
K07305	180.481002807617
K07359	239.240997314453
K07374	1260.39889836311
K07375	1418.06572210789
K07385	42.894100189209
K07390	659.863014221191
K07393	537.441297531128
K07399	9.51204991340637
K07407	142.44390130043
K07432	54.3098001480103
K07441	20.3285999298096
K07442	24.3458995819092

K07447	18.5873538851738
K07454	18.0709991455078
K07456	8.50493001937866
K07466	596.078094482422
K07478	3.46282005310059
K07497	56.4496632218361
K07498	10.3930997848511
K07512	10.7791996002197
K07513	1932.07416629791
K07541	61.7563600540161
K07542	68.9071400165558
K07555	18.699800491333
K07556	26.2693004608154
K07562	131.851596832275
K07565	21.1361999511719
K07566	268.791884422302
K07567	144.595993041992
K07573	67.698600769043
K07575	150.427001953125
K07579	43.3749008178711
K07583	9.72465991973877
K07604	0.681761026382446
K07735	48.9668006896973
K07739	274.798904418945
K07744	1066.30004882812
K07748	99.1720514297485
K07750	264.760851860046
K07759	25.6906026601791
K07760	209.479301452637
K07761	20.1138687729836
K07765	40.1065139770508
K07767	93.7394008636475
K07857	0.323383986949921
K07863	268.092971801758
K07870	248.331201553345
K07874	826.43098449707
K07875	115.610000610352
K07876	474.540985107422
K07877	287.187294006348
K07878	209.121994018555
K07881	78.065299987793
K07884	115.436996459961
K07887	385.365900039673
K07888	371.822799682617
K07889	426.766899108887
K07890	45.121898651123
K07893	442.975196838379
K07897	293.779998779297
K07901	340.635009765625
K07904	397.273696899414

K07905	397.273696899414
K07910	213.972593307495
K07933	39.5568990707397
K07936	620.566753208637
K07937	1485.47899532318
K07942	66.0055999755859
K07943	33.3111701011658
K07949	162.455001831055
K07950	162.455001831055
K07953	310.835998535156
K07955	387.199289321899
K07956	387.199289321899
K07964	72.0199701786041
K07975	268.092971801758
K07976	2305.05423259735
K07977	6100.57231998444
K08008	34.6738986968994
K08051	725.122985839844
K08054	752.355495452881
K08056	0.420026987791061
K08057	734.718470960855
K08059	141.641998291016
K08064	218.525268316269
K08065	301.961827278137
K08066	153.716898798943
K08070	726.334452629089
K08081	510.704417705536
K08094	17.3661994934082
K08099	29.7066993713379
K08101	12.1859798431396
K08136	6.26989984512329
K08137	138.766899108887
K08139	495.986160516739
K08145	98.0688095092773
K08149	46.8790988922119
K08150	151.188791275024
K08151	59.0989718437195
K08176	87.5596008300781
K08179	0.815407991409302
K08193	107.129590034485
K08202	5.40863287448883
K08232	367.940101623535
K08233	0.784802973270416
K08235	650.053874015808
K08237	124.049282550812
K08238	8.68982982635498
K08241	310.560946702957
K08242	23.051700592041
K08244	813.25540137291
K08245	831.985120415688

K08247	28.5615005493164
K08248	37.3450387716293
K08249	327.605997085571
K08252	34.0499000549316
K08253	77.0313410758972
K08266	68.5319905281067
K08269	100.352551102638
K08272	476.489208221436
K08282	424.972623467445
K08286	1225.84369027615
K08287	653.306364536285
K08288	139.583137333393
K08296	26.8971004486084
K08311	103.702003479004
K08316	33.4919013977051
K08332	293.968985319138
K08333	57.1051998138428
K08334	179.25719833374
K08337	132.188609838486
K08339	45.1338005065918
K08341	1523.71200561523
K08342	80.7847023010254
K08343	97.3187026977539
K08360	34.9982986450195
K08369	21.5317993164062
K08467	47.7243003845215
K08472	647.350777983665
K08486	227.541694641113
K08488	267.477996826172
K08489	99.1703996658325
K08490	39.8479995727539
K08492	43.4332008361816
K08493	133.973297119141
K08494	117.860000610352
K08495	175.895402908325
K08496	52.7966003417969
K08497	52.7416000366211
K08498	161.315200805664
K08500	161.315200805664
K08501	1.28218996524811
K08503	159.683389306068
K08504	839.777984619141
K08505	525.719970703125
K08506	192.147003173828
K08507	29.4422607421875
K08509	146.371002197266
K08511	575.147986412048
K08515	712.821997642517
K08516	307.276000976562
K08517	104.137001037598

K08518	15.0395796298981
K08573	20.2276000976562
K08577	20.2276000976562
K08578	20.2276000976562
K08580	20.2276000976562
K08592	19.4566993713379
K08596	6.68955993652344
K08597	67.4021987915039
K08653	94.7154998779297
K08657	5.89858984947205
K08658	193.138000488281
K08675	8.95942974090576
K08679	115.054899215698
K08681	67.1728973388672
K08695	191.056592941284
K08711	258.26739525795
K08712	13.2113003730774
K08727	23.7525005340576
K08730	31.3577501773834
K08734	16.0187304019928
K08735	80.020601272583
K08736	3.62541007995605
K08737	5.60299295186996
K08738	485.334312438965
K08739	7.7918039560318
K08740	9.94931983947754
K08741	0.965762972831726
K08742	171.108701705933
K08744	15.3933000564575
K08764	335.914001464844
K08769	245.654998779297
K08770	1625.28129196167
K08773	179.786003112793
K08775	3.0476188659668
K08776	100.640098571777
K08789	75.0233099460602
K08790	146.953596115112
K08803	72.7166118621826
K08817	45.5282001495361
K08818	313.540600776672
K08819	407.344369888306
K08823	21.7752902507782
K08825	228.112917900085
K08827	38.4712098836899
K08829	1700.61958599091
K08830	336.365504264832
K08832	81.8362998962402
K08835	81.5295649468899
K08838	5.73047006130219
K08839	10.8190210461617

K08849	26.2953205108643
K08850	8.52655029296875
K08851	24.9652299880981
K08852	172.265646934509
K08853	173.609001159668
K08856	356.552001953125
K08857	282.338079452515
K08864	12.693699836731
K08866	6.39369988441467
K08867	2.55550003051758
K08869	2528.97303652763
K08873	35.2199494838715
K08874	19.5145421624184
K08876	116.957698822021
K08880	9.60426044464111
K08882	0.316231995820999
K08892	6.50894021987915
K08900	173.794440984726
K08901	13.0423004627228
K08903	2.00205993652344
K08907	57.4758987426758
K08908	2.2749400138855
K08909	34.9233016967773
K08911	8.06713962554932
K08912	269.287764072418
K08913	269.287764072418
K08914	269.287764072418
K08915	79.1585006713867
K08916	72.8170013427734
K08917	138.804992675781
K08955	140.676498413086
K08956	125.097868919373
K08957	50.3193900585175
K08959	801.144916534424
K08960	860.359345912933
K08963	125.027599334717
K08967	151.942001342773
K08991	8.418869972229
K08997	109.537002563477
K08999	815.091888427734
K09008	169.100997924805
K09013	219.417198181152
K09014	213.175994873047
K09015	41.8384017944336
K09020	2.63583993911743
K09060	175.813529491425
K09122	15.7258200645447
K09131	43.2382011413574
K09140	45.9810981750488
K09187	26.9241008758545

K09191	91.0580024719238
K09201	21.2651996612549
K09228	21.2651996612549
K09250	178.369641065598
K09257	14.1241097450256
K09264	3171.76292091608
K09272	40.4567985534668
K09276	0.650139987468719
K09284	65.2024890780449
K09285	16.8475396633148
K09286	1682.91659474373
K09287	23.128999710083
K09291	41.9471802711487
K09313	67.3815002441406
K09338	1563.70388120413
K09367	0.979059994220734
K09377	522.409694671631
K09414	47.7738592028618
K09415	26.6669006347656
K09417	21.8799991607666
K09419	20.9631004333496
K09422	1342.16524958611
K09448	1422.10005283356
K09454	23.4548101425171
K09458	174.693678855896
K09480	121.811798095703
K09486	1080.77700483799
K09487	364.661987304688
K09489	152.31091594696
K09490	3169.48765861988
K09493	452.065200805664
K09494	170.452901646495
K09495	211.316898345947
K09496	31.7642993927002
K09497	179.709424853325
K09498	36.7680015563965
K09499	118.928497314453
K09500	69.8867888450623
K09502	944.171020507812
K09503	2299.85596001148
K09504	82.6983003616333
K09505	1014.00872039795
K09506	18.5960006713867
K09507	69.8376998901367
K09510	342.460597276688
K09511	69.8376998901367
K09512	2.07453989982605
K09514	2.07453989982605
K09515	69.8376998901367
K09516	54.4059982299805

K09517	70.2128982543945
K09518	193.094617843628
K09519	52.7359008789062
K09521	69.8376998901367
K09522	54.4203987121582
K09523	29.9679002761841
K09527	60.7058000564575
K09528	65.2425003051758
K09529	18.9662990570068
K09531	11.0956001281738
K09533	59.9228982925415
K09534	228.502504348755
K09540	489.865699768066
K09548	121.769996643066
K09549	289.358001708984
K09550	222.434997558594
K09553	372.9328956604
K09560	431.216493099928
K09561	89.6854019165039
K09562	43.3568000793457
K09564	17.1784992218018
K09565	682.108004331589
K09566	57.834400177002
K09567	1.82011997699738
K09569	753.297973632812
K09571	264.847232431173
K09580	605.96598815918
K09584	309.690023541451
K09587	3.96883988380432
K09588	80.5289001464844
K09590	5.24597012996674
K09595	877.602020263672
K09597	350.884994506836
K09598	156.847899436951
K09602	109.429000854492
K09613	39.9329986572266
K09645	47.3955001831055
K09646	52.8390598297119
K09648	26.327600479126
K09650	74.6815032958984
K09651	38.3082008361816
K09660	144.147994995117
K09667	230.779300689697
K09680	119.101398468018
K09699	121.541999816895
K09701	16.1056995391846
K09705	175.012195587158
K09710	131.136993408203
K09753	1682.49377185106
K09754	13.2881002426147

K09755	140.680420398712
K09756	7.2433500289917
K09761	11.6326999664307
K09773	22.3419404029846
K09775	86.9691009521484
K09828	4.48972988128662
K09833	51.357400894165
K09834	18.3020000457764
K09835	29.3181009292603
K09837	421.696196079254
K09838	13.8315000534058
K09839	69.1056003570557
K09840	757.780765533447
K09841	67.6712305545807
K09842	190.622599005699
K09843	40.0179901123047
K09872	1262.70466899872
K09873	340.841993808746
K09874	70.1205315589905
K09875	173.967800140381
K09880	808.864990234375
K09885	16.1846899986267
K09903	122.886659145355
K09919	92.8693008422852
K09955	148.088302612305
K10046	99.9224014282227
K10047	87.0839004516602
K10084	61.8337703943253
K10085	29.8016300201416
K10088	52.2714996337891
K10130	9.06620979309082
K10134	91.9854965209961
K10140	18.0313600301743
K10143	108.36371922493
K10144	720.52681350708
K10145	0.391525000333786
K10146	0.391525000333786
K10151	10.7552291154861
K10158	51.6637992858887
K10206	143.910995483398
K10251	143.495302200317
K10255	104.281101226807
K10256	498.975397109985
K10257	296.232886791229
K10258	416.058442115784
K10259	32.8880996704102
K10260	47.5485796928406
K10268	403.387385129929
K10290	48.2109985351562
K10293	34.5979804992676

K10295	136.872299194336
K10317	9.06767988204956
K10325	5.00630021095276
K10352	6.96324014663696
K10355	2752.28311920166
K10356	92.8415063619614
K10357	60.9194984436035
K10359	9.98461008071899
K10364	9.89959025382996
K10365	6.28869009017944
K10380	481.95112156868
K10389	18.6921005249023
K10393	333.500694274902
K10394	8.1080904006958
K10395	8.27917957305908
K10398	58.9452697038651
K10400	7.71132290363312
K10401	56.9114999771118
K10405	2.32592010498047
K10406	37.0151901245117
K10407	89.1596984863281
K10418	6.06362009048462
K10436	72.5820999145508
K10442	8.30263996124268
K10443	52.1128997802734
K10455	27.2071390151978
K10456	120.63200378418
K10457	16.7194995880127
K10458	62.5525016784668
K10464	81.5961990356445
K10469	30.6159992218018
K10477	23.5304503440857
K10521	67.8475036621094
K10523	62.6638011932373
K10525	179.511001586914
K10526	76.6980485022068
K10527	1823.3588886261
K10528	3.05868005752563
K10532	226.50341796875
K10536	117.975696563721
K10563	10.9111995697021
K10569	20.6203002929688
K10570	10.5347003936768
K10571	19.9428205490112
K10572	57.2112503051758
K10573	1604.82995605469
K10574	1604.82995605469
K10575	284.00700378418
K10576	282.79639339447
K10577	569.53099822998

K10578	347.463989257812
K10579	1943.39895629883
K10581	227.588113665581
K10583	50.539559841156
K10588	42.6166000366211
K10589	80.2765312194824
K10590	219.26189994812
K10591	48.0462007522583
K10592	260.881056308746
K10594	487.102996826172
K10595	39.2161908149719
K10597	178.348701477051
K10598	449.356488227844
K10599	46.3623008728027
K10604	56.3308982849121
K10606	19.7951799631119
K10609	358.831920981407
K10610	102.030571937561
K10614	72.4419021606445
K10615	72.4419021606445
K10629	201.604904174805
K10631	76.6415023803711
K10632	43.6804008483887
K10636	79.4001007080078
K10638	13.2178798019886
K10639	77.5440477132797
K10640	65.4673004150391
K10643	82.7958011627197
K10644	7.0717601776123
K10645	55.7316989898682
K10646	32.8714981079102
K10661	133.159400939941
K10666	121.602871417999
K10669	20.0683002471924
K10676	16.9553093910217
K10684	143.099597930908
K10685	57.6068019866943
K10688	112.717002868652
K10689	195.363006591797
K10690	1604.82995605469
K10691	32.6826305389404
K10695	50.959300994873
K10696	138.038799285889
K10703	59.9057912826538
K10704	10.777099609375
K10705	235.175003051758
K10707	44.109001159668
K10712	145.412163257599
K10717	489.72247505188
K10727	3.85122013092041

K10728	25.3549499511719
K10730	3.60838007926941
K10732	24.6830999851227
K10733	12.9245004653931
K10734	1.22456002235413
K10735	27.3265991210938
K10736	7.60259008407593
K10737	8.41361802816391
K10738	21.7765898406506
K10743	9.53592014312744
K10744	2.79305005073547
K10745	15.7720003128052
K10746	13.5086841583252
K10747	32.5256702899933
K10750	22.4744501113892
K10751	6.58273983001709
K10752	187.615737915039
K10753	30.9277992248535
K10754	44.9196996688843
K10755	65.826000213623
K10756	13.9022603034973
K10757	3.7225399017334
K10758	32.6567001342773
K10760	15.4462104439735
K10761	57.7386102676392
K10765	11.0777997970581
K10768	5.13495016098022
K10770	20.5371599197388
K10772	12.0172297954559
K10773	19.389095723629
K10775	32.1533391475677
K10777	4.76793992519379
K10779	20.4963307380676
K10781	1783.17680692673
K10782	312.483612060547
K10798	253.270161449909
K10799	71.7916111946106
K10802	6097.36545944214
K10803	34.4698778986931
K10807	4.86487007141113
K10808	48.6777000427246
K10827	122.04270863533
K10834	49.1776008605957
K10836	127.88484287262
K10838	6.33099985122681
K10839	1155.00681686401
K10840	243.763000488281
K10841	100.262378454208
K10842	45.0895195007324
K10843	176.706508755684

K10844	10.5601997375488
K10846	16.5872104167938
K10848	32.0454205274582
K10849	8.18175983428955
K10858	6.42071008682251
K10860	20.5532402992249
K10862	365.512588500977
K10863	56.8850002288818
K10866	48.4495493173599
K10867	6.74936008453369
K10870	4.50557994842529
K10871	21.1879795789719
K10872	4.16118001937866
K10875	30.1495304107666
K10877	8.05508041381836
K10878	44.8585386872292
K10879	1.44646900892258
K10884	9.60508024692535
K10885	4.48644018173218
K10886	17.6389000415802
K10891	8.3547140955925
K10895	3.25669598579407
K10896	5.39869502186775
K10899	11.4169998168945
K10900	5.15313005447388
K10901	13.6546002626419
K10902	5.85122013092041
K10903	25.7737193107605
K10907	10.4503002166748
K10908	6.47763013839722
K10949	528.963025093079
K10950	26.9691009521484
K10956	292.092405319214
K10960	108.317901611328
K10976	26.9691009521484
K10994	148.026459813118
K10999	2042.40552759171
K11000	309.600217819214
K11085	4.11003005504608
K11086	18.3346004486084
K11088	54.9875984191895
K11090	210.165901184082
K11092	127.964996337891
K11093	136.114001274109
K11094	506.89720916748
K11095	59.6800994873047
K11098	258.964996337891
K11099	192.720993041992
K11108	86.030403137207
K11111	3.25184011459351

K11126	4.98074996471405
K11128	11.6140003204346
K11129	123.171449184418
K11135	101.568405151367
K11136	3.13052999973297
K11137	17.8880004882812
K11145	38.5032005310059
K11147	131.675003051758
K11152	506.102567195892
K11153	271.394798278809
K11155	12.7018003463745
K11157	59.7714996337891
K11159	1139.28601074219
K11161	0.787840008735657
K11165	82.1877093315125
K11168	44.5591011047363
K11169	4.4198899269104
K11186	0.66994297504425
K11187	229.585998535156
K11188	17.8736991882324
K11228	319.650309443474
K11229	31.0829405784607
K11230	9.89966011047363
K11238	231.320999145508
K11247	114.061100006104
K11251	829.986074447632
K11252	558.463996887207
K11253	12.6515998840332
K11254	499.24388885498
K11262	111.657302856445
K11266	30.554500579834
K11267	182.484860658646
K11268	4.53337001800537
K11271	0.660016000270844
K11273	8.14848017692566
K11274	1.89358896017075
K11276	5.82986998558044
K11279	387.394385814667
K11290	49.5912017822266
K11291	1.86951798200607
K11292	204.453098297119
K11293	45.8877296447754
K11294	846.808517813683
K11303	78.1445999145508
K11308	54.1357002258301
K11314	57.8264999389648
K11320	8.54916000366211
K11322	17.9842703342438
K11324	54.3366012573242
K11338	6.93805980682373

K11339	33.0850475430489
K11341	69.827600479126
K11344	39.2742004394531
K11346	85.7476005554199
K11353	562.575012207031
K11362	36.6260986328125
K11363	191.005104064941
K11364	367.189598083496
K11366	67.9913084506989
K11367	154.462400436401
K11368	50.5713005065918
K11373	5.85221004486084
K11374	38.9174995422363
K11375	3.06030011177063
K11378	11.4073300361633
K11380	13.7842998504639
K11393	27.358699798584
K11404	42.9143981933594
K11407	133.67777711153
K11414	8.66205990314484
K11416	32.1115198135376
K11418	20.2369797229767
K11419	37.2634010314941
K11420	92.7137799263
K11422	19.3731298446655
K11423	53.6687002182007
K11424	63.0666999816895
K11426	59.7976698875427
K11430	38.4284901618958
K11433	10.504599571228
K11434	160.719497680664
K11436	11.4364004135132
K11437	33.7393989562988
K11438	7.71631002426147
K11439	118.499496459961
K11446	232.221745491028
K11449	99.330152451992
K11450	54.2810598611832
K11456	0.456779986619949
K11462	28.0356006622314
K11490	20.3750991821289
K11491	5.28923898935318
K11494	521.509907007217
K11498	63.9822406768799
K11507	50.2700996398926
K11517	65.2019009590149
K11518	30.9060001373291
K11527	1.80561995506287
K11547	4.16202020645142
K11548	30.2377996444702

K11557	215.074996948242
K11578	48.5541000366211
K11583	55.9790000915527
K11584	274.086273193359
K11592	80.7153776884079
K11593	745.466901898384
K11594	103.724296569824
K11596	864.882971167564
K11599	277.031005859375
K11600	38.339298248291
K11642	4.34315013885498
K11643	42.0739691257477
K11644	359.088998794556
K11646	9.59492015838623
K11647	52.6877603530884
K11649	161.715970516205
K11650	29.7914009094238
K11652	21.7266006469727
K11654	52.8216886520386
K11655	4.90632009506226
K11657	9.67483043670654
K11659	39.9954986572266
K11662	8.29638957977295
K11663	246.702239751816
K11664	16.8598003387451
K11665	290.567759037018
K11666	70.6772003173828
K11667	21.6037998199463
K11671	15.0241301059723
K11672	14.6169004440308
K11673	12.9465999603271
K11684	50.8425512313843
K11699	31.7520883083344
K11713	43.4123001098633
K11714	116.053802490234
K11717	117.545001983643
K11718	42.6147994995117
K11721	14.9450998306274
K11723	60.9080991744995
K11745	67.6349391937256
K11747	67.6349391937256
K11752	66.9414005279541
K11755	90.5213012695312
K11757	19.5228996276855
K11770	35.9659996032715
K11778	96.8178291320801
K11786	10.0170001983643
K11789	19.7660007476807
K11793	54.8333015441895
K11798	9.91829013824463

K11799	20.7798004150391
K11801	569.603187561035
K11804	1036.12010288239
K11805	17.7444000244141
K11806	116.374996185303
K11808	12.4597997665405
K11816	7.47018003463745
K11817	197.619628787041
K11819	166.582992553711
K11820	7.74402022361755
K11821	12.2242795825005
K11824	50.3559989929199
K11827	325.532005310059
K11835	63.8904113769531
K11836	69.3021984100342
K11838	541.385652542114
K11839	13.1330995559692
K11840	0.723610997200012
K11841	88.1218976974487
K11842	94.3295993804932
K11843	131.247798919678
K11844	4.8723201751709
K11851	5.14551019668579
K11855	93.811888217926
K11858	52.0331983566284
K11863	28.0583000183105
K11864	50.2773017883301
K11866	721.954854726791
K11868	564.690113544464
K11876	38.4280014038086
K11877	32.5957000255585
K11883	101.137699127197
K11884	79.0543975830078
K11885	88.5619964599609
K11886	55.4392595291138
K11927	46.5917301177979
K11968	299.805997371674
K11971	81.1952991485596
K11972	17.7012004852295
K11975	8.89369010925293
K11979	42.8068008422852
K11982	824.91420674324
K11984	31.5709991455078
K11996	46.8263993263245
K12115	168.385499000549
K12116	168.385499000549
K12117	168.385499000549
K12118	244.081001281738
K12119	244.081001281738
K12120	159.51487827301

K12121	159.51487827301
K12122	159.51487827301
K12123	159.51487827301
K12124	197.944801330566
K12125	198.022994995117
K12126	1.31269001960754
K12127	41.2566998004913
K12128	10.1336002349854
K12129	135.875299453735
K12130	26.7765793800354
K12132	297.489796638489
K12135	256.053517103195
K12162	640.059020996094
K12163	754.15279006958
K12164	80.834098815918
K12169	28.2616996765137
K12173	36.8286018371582
K12175	141.391101837158
K12176	156.293322205544
K12177	105.436996459961
K12178	25.229700088501
K12179	73.2984008789062
K12180	84.9792022705078
K12181	67.0147018432617
K12183	26.3038997650146
K12184	38.7419013977051
K12185	126.488304138184
K12188	37.3941993713379
K12189	85.9160995483398
K12190	26.3122005462646
K12191	289.476989746094
K12192	154.087005615234
K12194	387.590087518096
K12195	175.149993896484
K12196	352.149192810059
K12197	435.352996826172
K12198	282.130996704102
K12199	48.9954986572266
K12200	60.6395988464355
K12231	30.8963003158569
K12232	3.97305989265442
K12235	18.1439003944397
K12236	22.7242002487183
K12251	85.1764984130859
K12256	129.938697814941
K12259	6.70276021957397
K12261	178.863998413086
K12271	9.69076979160309
K12272	50.2634010314941
K12275	105.005996704102

K12309	1986.32302461565
K12311	132.939319610596
K12343	165.664503097534
K12345	3.07941007614136
K12347	127.88500213623
K12349	56.2650985717773
K12355	324.260987281799
K12356	0.768660008907318
K12373	1170.78920078278
K12375	2.32260990142822
K12382	1497.33965110779
K12385	81.8605003356934
K12386	342.734008789062
K12391	35.1057014465332
K12392	390.205501556396
K12393	96.109001159668
K12396	11.6365599632263
K12397	37.1494607925415
K12398	79.774299621582
K12399	21.5585298538208
K12400	40.2422981262207
K12401	37.4476013183594
K12402	125.869003295898
K12403	201.337997436523
K12405	390.466003417969
K12418	17.2206001281738
K12446	38.5738010406494
K12447	57.0704002380371
K12448	25.2378997802734
K12449	416.285003662109
K12450	242.974395751953
K12451	129.861999511719
K12460	67.4271297454834
K12462	27.054519534111
K12467	42.894100189209
K12469	163.95369720459
K12471	410.348912715912
K12476	163.95369720459
K12477	163.95369720459
K12479	115.799200057983
K12483	163.95369720459
K12486	135.873001098633
K12489	165.599899291992
K12492	305.470708847046
K12493	643.159472584724
K12501	52.7504801750183
K12502	83.5844788551331
K12524	6539.12200164795
K12580	64.2298984527588
K12581	253.155996322632

K12585	42.2875995635986
K12586	6.69590997695923
K12587	81.9711990356445
K12591	86.0956506729126
K12593	39.1316986083984
K12598	119.743499755859
K12599	16.2288899421692
K12600	63.3849582672119
K12602	41.1440010070801
K12603	144.12563085556
K12604	19.8568992614746
K12605	69.8936309814453
K12606	75.6773996353149
K12607	28.9878101348877
K12608	72.1559991836548
K12611	59.6483306884766
K12613	47.7350997924805
K12614	157.467002868652
K12616	25.9762992858887
K12617	101.079200744629
K12619	225.389962464571
K12620	139.880996704102
K12621	277.910701751709
K12623	148.225997924805
K12624	60.7932014465332
K12627	72.0030975341797
K12640	5.24597012996674
K12655	59.5873985290527
K12657	43.3804016113281
K12662	80.8429985046387
K12663	37.1293983459473
K12666	135.410598754883
K12667	105.930999755859
K12668	301.816986083984
K12669	31.1517798900604
K12670	202.97900390625
K12733	38.2697982788086
K12734	34.9990005493164
K12735	11.1735301017761
K12736	19.0419998168945
K12737	17.755199432373
K12741	356.292136669159
K12742	430.763895273209
K12795	140.542007446289
K12811	22.9743399620056
K12812	349.325096130371
K12813	26.5991291999817
K12815	26.407600402832
K12816	65.0679111480713
K12817	33.4813995361328

K12818	91.8642597198486
K12819	272.412002563477
K12820	249.796183586121
K12821	134.110919952393
K12822	119.853202819824
K12823	414.688196182251
K12824	16.5919990539551
K12825	64.8738794326782
K12826	78.7101974487305
K12827	286.975003242493
K12828	36.1497001647949
K12829	170.882099151611
K12830	15.6691999435425
K12831	157.144100189209
K12834	125.166000366211
K12835	61.5649013519287
K12836	84.9875030517578
K12837	160.889032363892
K12839	189.279899597168
K12840	65.5711975097656
K12842	91.8022003173828
K12843	114.614570975304
K12844	102.842098236084
K12845	208.432998657227
K12847	73.4997978210449
K12848	135.943496704102
K12850	47.7974014282227
K12852	72.5176992416382
K12854	81.9771106243134
K12855	41.8570199012756
K12856	121.24179649353
K12857	80.5945014953613
K12858	25.1949996948242
K12859	677.162874221802
K12860	81.7496013641357
K12861	49.244499206543
K12862	45.7088012695312
K12863	203.333999633789
K12864	76.9909710884094
K12865	26.6361999511719
K12867	8.3530797958374
K12868	148.720993041992
K12869	16.4440994262695
K12871	43.723201751709
K12872	54.0424003601074
K12874	8.38376045227051
K12875	76.5832977294922
K12876	126.704002380371
K12877	115.46900177002
K12878	101.940900802612

K12879	36.3910999298096
K12880	83.7764015197754
K12881	663.230907440186
K12882	27.2564001083374
K12883	77.2733993530273
K12884	0.870055973529816
K12886	0.891902983188629
K12888	0.586812019348145
K12890	64.8002500534058
K12891	124.930999755859
K12893	0.335251003503799
K12897	114.395980358124
K12898	485.301706433296
K12929	16.1016998291016
K12930	3.7225399017334
K12946	34.6137008666992
K12947	262.280502319336
K12948	338.731994628906
K12960	63.268798828125
K12968	4.73669004440308
K13024	5.5227198600769
K13025	1181.93948459625
K13030	121.372918486595
K13034	736.505344927311
K13035	37.5693998336792
K13044	127.280002593994
K13051	54.7860994338989
K13064	32.1533391475677
K13065	484.268392324448
K13066	183.329803466797
K13067	10.7613000869751
K13068	6.54441022872925
K13070	194.632995605469
K13071	133.281744241714
K13076	177.804629921913
K13082	194.233359098434
K13091	191.49978017807
K13093	61.2864499092102
K13094	181.679319381714
K13095	89.3013401031494
K13096	271.093994140625
K13098	0.632463991641998
K13099	59.4505882263184
K13100	64.9902300834656
K13101	49.2925987243652
K13102	11.8535003662109
K13103	34.7255010604858
K13104	28.8006000518799
K13105	15.8936004638672
K13106	33.8278007507324

K13107	26.7301006317139
K13108	50.9266014099121
K13109	40.8540000915527
K13110	162.589099884033
K13111	36.4497985839844
K13114	26.5648994445801
K13115	260.349006652832
K13116	70.2031021118164
K13117	11.5455102920532
K13118	22.9461994171143
K13119	49.2509002685547
K13121	80.8059997558594
K13123	5.09294986724854
K13124	13.4069004058838
K13126	604.621078073978
K13127	84.2877006530762
K13128	50.1758995056152
K13130	19.9841003417969
K13136	390.633012771606
K13137	276.586006164551
K13140	5.97939318418503
K13144	29.1938220709562
K13146	10.0728998184204
K13148	470.264361351728
K13150	23.8689000606537
K13151	104.368999481201
K13153	41.7772212028503
K13154	8.00152015686035
K13155	20.6051998138428
K13156	7.50321000814438
K13157	28.8145308494568
K13158	3.02259504795074
K13159	0.767696976661682
K13160	136.141500473022
K13161	327.605504989624
K13162	883.745904445648
K13168	166.262298583984
K13173	12.9764995574951
K13174	87.2024993896484
K13175	33.4928016662598
K13176	51.7481994628906
K13179	119.088171958923
K13181	19.1201992034912
K13182	349.325096130371
K13183	191.078002929688
K13187	28.6930999755859
K13190	4.954430103302
K13192	59.5966787338257
K13194	14.9723303318024
K13195	209.105894088745

K13199	697.787506699562
K13201	1682.9147105217
K13206	43.6786003112793
K13207	118.974901199341
K13210	417.985207557678
K13211	38.3795199394226
K13212	75.8194198608398
K13213	2.33916103839874
K13216	46.4752583503723
K13217	287.978110551834
K13220	117.033996582031
K13223	43.85471534729
K13225	43.85471534729
K13229	148.480501174927
K13230	3932.14314937592
K13232	67.0098037719727
K13237	190.365997314453
K13248	32.7911987304688
K13249	71.1529006958008
K13250	116.832000732422
K13254	47.5475006103516
K13278	11.7938003540039
K13279	0.66994297504425
K13280	209.20280456543
K13288	38.5168991088867
K13289	882.455107688904
K13335	30.0836498737335
K13336	53.2044982910156
K13337	72.6035995483398
K13338	26.0992197990417
K13339	77.3843661546707
K13341	19.9708003997803
K13342	157.273204803467
K13343	150.105380535126
K13345	40.0871000289917
K13346	14.1165500879288
K13347	18.544900894165
K13348	359.361799240112
K13353	78.7853012084961
K13354	555.573369503021
K13356	1865.44498443604
K13366	7.40527009963989
K13367	839.17501449585
K13379	98.2372970581055
K13412	587.52729010582
K13413	96.7597026824951
K13414	180.56770324707
K13415	659.410616159439
K13416	1827.51141625643
K13417	332.212726891041

K13418	1827.51141625643
K13420	1118.95483785868
K13421	284.150002479553
K13422	141.932768344879
K13424	222.417538881302
K13425	22.1366505622864
K13427	25.9671001434326
K13428	76.5511944890022
K13429	224.704956293106
K13430	1777.2592793107
K13431	284.680999755859
K13447	91.3569670319557
K13448	1835.64999747276
K13449	168.413592815399
K13456	190.369860172272
K13457	397.695120453835
K13458	36.2622985839844
K13459	387.032578468323
K13462	157.588299751282
K13463	92.2860012054443
K13464	958.16620850563
K13473	4.55663013458252
K13484	54.1455001831055
K13493	16.5604499578476
K13495	47.0656250715256
K13496	291.43242764473
K13506	90.2499008178711
K13508	112.88257932663
K13510	107.538887023926
K13511	78.1443977355957
K13513	22.1951094865799
K13519	126.304697990417
K13523	106.747940063477
K13525	956.536292791367
K13528	41.988899230957
K13535	114.973999023438
K13544	94.8967094421387
K13545	24.217399597168
K13566	88.1380996704102
K13577	6.38748979568481
K13600	16.0720996856689
K13606	594.219391822815
K13621	134.192380070686
K13628	630.680152773857
K13648	604.153227567673
K13667	30.6319804191589
K13679	78.5214996337891
K13680	247.318042516708
K13681	72.099761724472
K13691	7.74402022361755

K13692	39.830470085144
K13696	10.038800239563
K13711	59.7308006286621
K13717	67.2868406772614
K13719	244.455993652344
K13726	7.36632013320923
K13728	17.2553005218506
K13730	373.47203540802
K13754	54.2423710823059
K13783	56.1392711400986
K13789	244.851296186447
K13800	46.1828002929688
K13811	147.876637458801
K13832	34.323299407959
K13863	141.407697677612
K13864	141.407697677612
K13865	141.407697677612
K13917	177.442596435547
K13946	203.481874465942
K13947	49.006178855896
K13950	14.1733399629593
K13951	857.520641833544
K13963	86.1330032348633
K13979	2243.58216714859
K13981	323.246002197266
K13982	44.2182306051254
K13983	5.30961990356445
K13984	82.7816009521484
K13989	327.222649335861
K13993	468.719190120697
K13998	93.9628064036369
K14001	50.0989990234375
K14002	52.562801361084
K14003	110.10120010376
K14004	252.242595672607
K14005	40.9099006652832
K14006	188.868549346924
K14007	149.011003494263
K14009	117.586011886597
K14011	34.3126983642578
K14012	227.030598640442
K14015	46.5060997009277
K14016	199.580169677734
K14018	43.481530547142
K14026	49.2695999145508
K14050	142.822101593018
K14066	76.1602020263672
K14077	12.1843004226685
K14079	21.9556894302368
K14085	344.60400390625

K14137	53.941890001297
K14153	15.9813003540039
K14156	63.1451988220215
K14157	67.0251007080078
K14164	6.56794023513794
K14165	221.80549621582
K14168	20.5228004455566
K14169	10.745400428772
K14172	96.4599990844727
K14190	237.566492080688
K14191	88.8238003253937
K14207	261.730281591415
K14209	22.7632691860199
K14213	103.966003417969
K14267	163.664402961731
K14270	11.7655799388885
K14288	6.92852020263672
K14289	31.013920545578
K14290	64.7269667983055
K14292	28.83899974823
K14293	44.6600208282471
K14294	35.0172996520996
K14295	18.6331996917725
K14297	50.5621485710144
K14298	31.0645399093628
K14299	44.4747009277344
K14300	10.7161998748779
K14301	22.8290004730225
K14303	23.6684601306915
K14304	62.6593017578125
K14305	31.4229898452759
K14308	27.1742000579834
K14309	36.5577993392944
K14310	10.950400352478
K14311	3.44406008720398
K14312	19.2741594314575
K14313	34.7840995788574
K14314	26.620593726635
K14315	28.2680997848511
K14317	16.5442295074463
K14318	21.4977498054504
K14319	34.1128997802734
K14320	9.3779296875
K14321	10.6155996322632
K14324	59.6750984191895
K14325	58.8652992248535
K14326	122.947710514069
K14327	118.252955138683
K14328	34.1562004089355
K14347	118.852703094482

K14360	38.160701751709
K14376	167.218199491501
K14396	171.114097595215
K14397	83.9821615219116
K14399	18.108571767807
K14401	9.50502991676331
K14402	116.973598480225
K14403	46.1587982177734
K14404	48.4459767341614
K14405	6.62330007553101
K14406	12.5728998184204
K14407	49.0703010559082
K14408	11.5999002456665
K14409	73.6122970581055
K14411	1041.22237253189
K14412	12.5362997055054
K14413	219.102467536926
K14416	568.966163635254
K14423	127.629997253418
K14424	61.566798210144
K14427	478.528694152832
K14431	291.074239015579
K14432	76.0225992202759
K14439	85.1827993392944
K14440	5.54228973388672
K14442	232.048613548279
K14445	138.714019298553
K14452	124.707504272461
K14453	132.0002784729
K14455	105.698997497559
K14457	448.167499542236
K14484	2406.5021815896
K14485	1117.70731163025
K14486	1481.64452970028
K14487	233.799659490585
K14488	88.4017918109894
K14489	169.317752480507
K14490	132.210541248322
K14491	256.373323738575
K14492	257.903510630131
K14493	430.578319072723
K14494	844.500777065754
K14495	54.5525016784668
K14496	73.0670991539955
K14497	1007.19859218597
K14498	633.612936019897
K14500	208.881110787392
K14501	1.04407000541687
K14502	605.863117218018
K14503	130.134016990662

K14504	227.899208068848
K14506	154.399898529053
K14508	144.644500732422
K14509	139.934101104736
K14510	529.421504974365
K14512	253.400001525879
K14513	135.506198883057
K14514	783.966347694397
K14515	213.954049825668
K14516	0.991460025310516
K14521	9.33782005310059
K14525	61.99289894104
K14535	133.143583297729
K14536	7.64197015762329
K14537	67.0361022949219
K14538	83.7753982543945
K14539	78.7326984405518
K14540	37.5661010742188
K14544	7.29538011550903
K14545	114.524002075195
K14546	100.474500656128
K14548	205.502722263336
K14550	29.8445737957954
K14552	8.47628021240234
K14553	19.9193992614746
K14554	18.4666004180908
K14555	16.1487703323364
K14556	51.4778995513916
K14557	23.9640007019043
K14558	367.871800422668
K14560	27.3292999267578
K14561	51.0838012695312
K14563	90.2993011474609
K14564	100.319999694824
K14565	33.1921005249023
K14566	131.012939214706
K14567	22.9745006561279
K14568	26.2285003662109
K14569	46.1806888580322
K14570	107.535563468933
K14571	89.7533016204834
K14572	22.7392107248306
K14573	376.361463904381
K14574	1871.9061768055
K14575	13.7758896350861
K14606	35.2371997833252
K14609	21.5466003417969
K14610	20.9572501182556
K14611	628.555661916733
K14617	192.494400024414

K14638	1041.07945477962
K14640	0.99974399805069
K14641	196.13920211792
K14648	242.910003662109
K14649	19.1441402435303
K14652	153.327272415161
K14662	12.1010999679565
K14664	386.96390247345
K14674	188.400700569153
K14677	78.8534984588623
K14682	27.1473999023438
K14685	17.3348007202148
K14686	158.645567893982
K14689	86.8258717656136
K14694	59.0772018432617
K14696	37.2247009277344
K14704	22.8729109764099
K14708	8.62538003921509
K14709	12.746570110321
K14713	155.511302947998
K14721	40.9819997549057
K14724	379.040421724319
K14726	44.8623008728027
K14729	2.57593989372253
K14753	80.6384011507034
K14758	801.144916534424
K14759	0.919018000364304
K14765	38.4650993347168
K14766	14.9979000091553
K14767	64.6688995361328
K14768	22.3423004150391
K14769	81.1410980224609
K14771	10.5269002914429
K14772	11.8219002485275
K14774	120.249200820923
K14775	101.899569034576
K14776	135.607597351074
K14777	137.508403778076
K14778	20.0466995239258
K14779	3.94323992729187
K14780	21.2199101448059
K14782	41.8283996582031
K14784	106.884002685547
K14786	10.600700378418
K14787	15.5804004669189
K14788	14.5959997177124
K14790	48.8336200714111
K14791	114.619501113892
K14792	21.0572501420975
K14793	14.0789003372192

K14794	52.3620798587799
K14795	81.9654006958008
K14797	51.8620986938477
K14798	19.0104007720947
K14799	104.097818851471
K14800	90.5419006347656
K14801	50.4083003997803
K14802	70.5910696387291
K14803	276.954986572266
K14805	22.8691005706787
K14806	30.3383007049561
K14807	24.1503000259399
K14808	24.8420405387878
K14809	859.722249031067
K14810	76.1594009399414
K14811	43.3644981384277
K14815	115.966003417969
K14816	82.3928985595703
K14818	21.3470993041992
K14819	247.513103485107
K14820	87.2732009887695
K14821	32.3913295269012
K14822	91.8033981323242
K14823	46.679500579834
K14824	43.2444000244141
K14825	144.184001922607
K14826	24.3404898643494
K14827	10.773099899292
K14829	209.155570626259
K14830	3.15430998802185
K14831	31.5946006774902
K14832	42.571400642395
K14833	22.2823506593704
K14834	19.1982097625732
K14835	158.469923019409
K14837	213.660483956337
K14838	122.606026232243
K14839	32.4874000549316
K14840	71.0268020629883
K14841	17.3492501974106
K14842	80.3932037353516
K14843	251.157402038574
K14844	116.099996566772
K14845	27.8283996582031
K14846	102.769298553467
K14847	26.4123001098633
K14848	51.9692993164062
K14849	117.818300247192
K14850	42.351001739502
K14851	67.0308990478516

K14852	71.3385009765625
K14855	44.5131015777588
K14856	34.7295999526978
K14857	23.4074001312256
K14859	15.1049003601074
K14861	620.295547485352
K14863	108.5482006073
K14864	46.1864013671875
K14944	334.265991210938
K14945	446.272703170776
K14948	534.641498565674
K14950	113.739402770996
K14961	11.9743995666504
K14962	43.6746888160706
K14963	177.084595680237
K14964	24.1823997497559
K14966	261.568881988525
K14972	5.26541996002197
K14977	35.6286890506744
K15014	188.059497833252
K15015	324.864417433739
K15026	68.9071006774902
K15027	50.2273006439209
K15028	243.570999145508
K15029	29.171199798584
K15030	65.8124008178711
K15031	7.79570007324219
K15032	149.887030363083
K15033	29.9032001495361
K15040	644.908201217651
K15043	441.860733747482
K15044	26.0979995727539
K15046	89.8577907085419
K15047	38.1208992004395
K15053	27.8572998046875
K15071	83.2972984313965
K15074	22.2395992279053
K15075	3.32413005828857
K15078	642.951090633869
K15082	30.3311996459961
K15083	107.983720779419
